# Green synthesis of *Illicium verum*-derived novel semiconducting Ag/Fe/Cu-trimetallic nanocomposites: A dual-functional platform for selective detection of pharmaceutical compounds and light-independent degradation of organic dyes

**DOI:** 10.1039/d6ra02869a

**Published:** 2026-05-18

**Authors:** Ambreen Zia, Ziana Manzar, Syed Nawazish Ali, Syeda Farah Bukhari, Imran Malik

**Affiliations:** a Department of Chemistry, University of Karachi Karachi-75270 Pakistan syed.nawazish@gmail.com snali@uok.edu.pk; b Department of Chemistry, NED University of Engineering and Technology Karachi-75270 Pakistan; c Department of Basic Science (Chemistry), Imam Abdulrahman Bin Faisal University Dammam Saudi Arabia

## Abstract

In the present work, phytochemicals derived from the extract of *Illicium verum* fruit have been used for the first time as capping, stabilizing and reducing agents to synthesize pH and thermally stable silver-, iron- and copper-based trimetallic nanocomposites (Ag/Fe/Cu-TNAs). Characterization was carried out by multiple spectroscopic and imaging techniques. Nanocomposites were found to be spherical in shape with an average size of 60 nm by scanning electron microscopy (SEM) and atomic force microscopy (AFM). The presence of all three metals was evident from energy-dispersive X-ray spectroscopic analysis (EDS). X-ray diffraction (XRD) revealed a crystallite size of 17.83 nm. Shifts in the peaks of Fourier transform infrared (FTIR) spectra and the appearance of a broad peak between 250 and 400 nm in the UV-visible spectrum also provided convincing evidence for the formation of nanocomposites. An optical band gap of 3.8 eV indicated the semiconducting nature of the nanocomposites. This research represents the first demonstration of trimetallic nanocomposites as green and cost-effective nanosensors for the detection of an important pharmaceutical residue, levocetirizine. Levofloxacin was also detected effectively at a lower concentration. The limit of detection values for levofloxacin and levocetirizine were found to be 4.43 µM and 5.49 µM, respectively. The stoichiometric ratio and binding constant values for levofloxacin (2 : 3, 1.139 × 10^4^ M^−1^) and levocetirizine (1 : 4, 1.153 × 10^5^ M^−1^) revealed the strong interactions between the nanocomposites and the drug molecules, enabling them to serve as distinctive nanosensors in tap and well water. Furthermore, the nanocomposites were utilized to evaluate their catalytic potential against methyl orange dye by UV-visible spectroscopy in the absence of light. The findings revealed that the rate of degradation was substantially enhanced (93.48% in 2 h) in the presence of nanocomposites as compared to bare H_2_O_2_ (33% in 2 h). Our study explores the potential use of semiconducting Ag/Fe/Cu-TNAs as bio-sensors and catalysts for promising and viable applications, especially in wastewater treatment, a step forward to meet the 17 Sustainable Development Goals (SDGs).

## Abstract 2

*Illicium verum* extract-based approach for trimetallic nanocomposites with sustainable potential in wastewater treatment.

## Graphical abstract

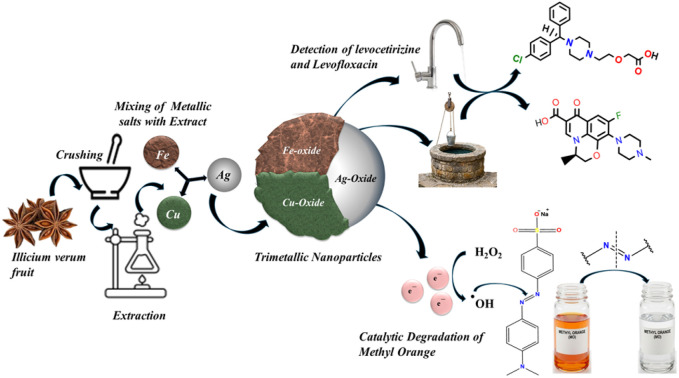

## 1. Introduction

The extensive pharmaceutical contamination of aquatic ecosystem has emerged as a growing global challenge, which threatens the ecological sustainability and human health. Elevated concentrations of pharmaceutical drugs have been detected in shallow water, drainage channels and sewage effluents because rising consumption of pharmaceuticals leads to the discharge of unmetabolized organic compounds into waste water.^1,2^ Antihistamine- and fluoroquinolone-based drugs pose serious health concerns, as they cause damage at the cellular level if left untreated.^3,4^ Levocetirizine is the third-generation antihistamine drug that binds with histamine receptors and suppresses their activity. It is used as an anti-allergic agent to treat various allergic diseases such as chronic urticarial and rhinitis.^5,6^ Levofloxacin (second-generation antibiotic) is employed for treating infections in the pulmonary, excretory and integumentary systems in human beings.^7^ However, extensive use of these medications implies serious health issues such as upper respiratory tract infections, temporary gastrointestinal dysfunctions, aggravations of allergies,^8^ hepatotoxicity, seizures, central nervous system excitation and ocular complications.^9^ Furthermore, disposal of pharmaceutical pollutants in the environment causes the emergence and development of drug-resistant bacteria and adversely interrupts the micro-ecological balance.^10,11^ Additionally, organic dyes are one of the major components present in the discharge of textile, food, leather and cosmetic industries that cause harm to aquatic as well as terrestrial life.^12^ Azo dyes such as methyl orange are widely used because they are readily available and cheap. Therefore, these dyes sustain in the environment over an extended time period and impose severe health impairments including seizures, nausea, gastrointestinal upset and tissue necrosis.^13^ Several methods have been developed for the waste water treatment including electrochemical,^14,15^ colorimetric,^16^ fluorometric^17^ and HPLC-driven chromatographic techniques.^18^ Recently, various nanomaterials have been employed as effective treatment agents. Among these, zinc-oxide nanorods,^19^ BaFe_2_O_4_,^20^ methionine-conjugated CaO,^21^ TiO,^22^ Cobalt,^23^ NiO,^24^ and Ag/AgO^25^ nanoparticles are being widely used.

Scientific literature has revealed very limited data about the detection of drugs, especially levocetirizine (Table 1).^14,26–33^ Conversely, a majority of these techniques lack adequate sensitivity, have a narrow scope and require expensive instruments with challenging analytical methods and highly skilled technicians, while the chemically synthesized nanomaterials involve the consumption of hazardous chemicals, which also increases the health risk.^34^ This drives the necessity for the development of affordable, simple-to-use materials and methods, which can be utilized without harming the environment and can be implemented to perform various tasks effectively.

**Table 1:** Recent advances in the detection of levocetirizine and levofloxacin

S. No		Mode of sensing	Sensor	Advantages	Disadvantages
1		Electrochemical	Potentiometric micro sensor	Fast response	Complex method
2	Levocetirizine	Electrochemical	Carbon paste electrode	High sensitivity	Complex method of synthesis
3		Chromatographic	LC-MS	High specificity and sensitivity	High cost and complex instrumentation
4		Electrochemical	MnTMIPP on indium tin oxide (ITO)	High selectivity and sensitivity	Complex method and chances of ITO degradation
5		Fluorescence	Self-healing Au-nanoclusters/PVA hydrogel	Self-repairing and high sensitivity	Chemical method of synthesis
6	Levofloxacin	Electrochemical	Co@CaHPO	High sensitivity	High ionic strength affects sensitivity
7		Potentiometric	Electro-polymerizing polyaniline on a glassy carbon electrode	Rapid technique	Limited sensitivity and chemical method of synthesis
8		Electrochemical	Poly(Pyrogallol red) on GCE	Stable for 3 weeks	Required strictly controlled pH (optimum 6.0)
9		Mass spectrometric	LC-MS/MS	Traced detection in human serum	Requires highly trained staff and expensive infrastructure

Over the recent years, nanomaterials that are classified as monometallic, bimetallic and multi-metallic systems have emerged widely to address these challenges. By combining these nanoparticles, new properties or synergistic effects are usually showcased as compared to individual entities.^35^ Due to this reason, the synthesis of trimetallic nanoparticles has become a topic of greater interest among the scientists as it contains intensified biological activities, catalytic potential, increased sensitivity, selectivity and stability. In comparison with mono- and bi-metallic nanoparticles, trimetallic nanoparticles possess extensively branched structures and properties of three metals.^36–38^ Therefore, their unique characteristics enable them to be used in numerous applications such as dye degradation,^39^ gas sensing,^40^ hydrogenation of acetylene,^41^ drug delivery^42^ and semiconductors^43^ in electronic devices. It is well established that the nanoparticle morphology plays a critical role in determining the reactivity and catalytic efficiency, with spherical structures commonly reported due to their thermodynamic stability and uniform surface characteristics.^44^ We focused the synthesis of Ag–Fe–Cu trimetallic nanoparticles with a spherical shape to develop systems of environmental remediation. Green synthesis of trimetallic nanoparticles signifies a cutting-edge strategy, which provides an innovative, sustainable and cost-effective approach. It also eliminates the liberation of toxic chemicals into the environment. Various bio-reducers such as plants, algae, yeast, bacteria and fungi have been employed to synthesize trimetallic nanoparticles.^45^ Among these, plants are the most selected route, which are rich in phenolic compounds acting as reducing agents. To date, there is very limited research for the synthesis of trimetallic nanoparticles that may be directly related to the corresponding extract composition.^46^ In the present research, phytochemicals derived from the extract of *Illicium verum* fruit were utilized as capping, reducing and stabilizing agents for the first time. *Illicium verum* also known as star anise is a traditional Chinese medicine which possesses potential antibacterial, antifungal and anti-inflammatory activities.^47^ The fruit of *Illicium verum* is an easily available spice and used in food; therefore, it can be used as an economically viable plant-based reducing agent. The objective of this research was to synthesize multifunctional and resource-efficient semiconducting trimetallic nanocomposites for monitoring pharmaceutical pollutants along with the efficient catalytic degradation of water-dispersible dyes without causing harm to the environment.

## 2. Results and discussion

### 2.1 Characterization of Ag/Fe/Cu-TNAs

#### 2.1.1 SEM and AFM analysis

**Fig. 1:**
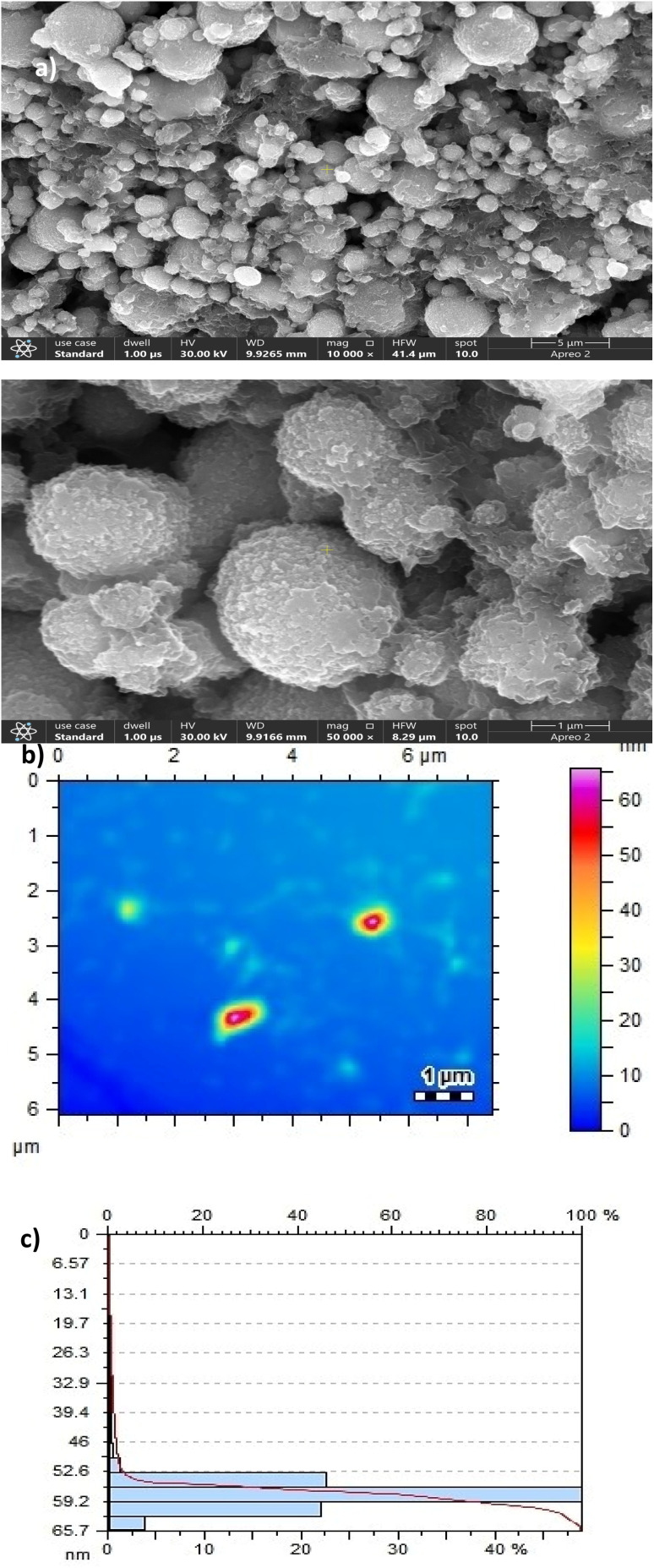
(a) SEM analysis (b) AFM analysis and (c) size distribution histogram of Ag/Fe/Cu-TNAs.

The surface morphology of Ag/Fe/Cu-TNAs was determined by SEM analysis (Fig. 1a), which represents that the particles are spherical in shape while some are agglomerated. The presence of some individual particles shows that agglomeration may occur during the process of nucleation and drying due to inter-particle attraction. The size of Ag/Fe/Cu-TNAs, ascertained from AFM analysis, was found to be in the range of 50–66 nm (Fig. 1b). Histogram (Fig. 1c) shows that particles with 60 nm diameter are present in higher concentrations. Low polydispersity was confirmed by the decreasing trend of the logarithmic plot, and the point of coincidence validates the maximum effective diameter of 60 nm.

#### 2.1.2 EDS analysis

**Fig. 2:**
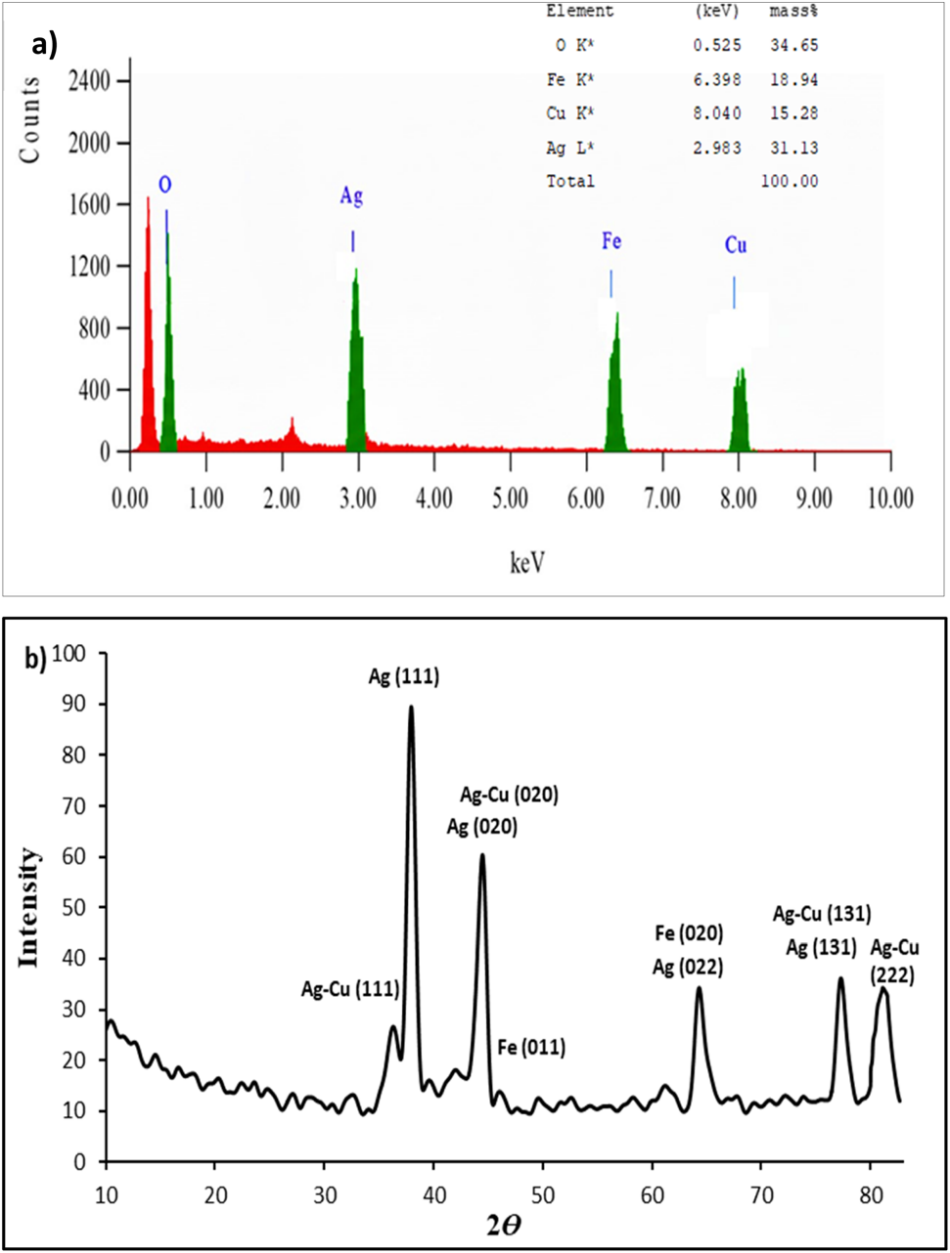
(a) EDS spectrum and (b) XRD pattern of Ag/Fe/Cu-TNAs.

EDS analysis (Fig. 2a) illustrates the elemental composition of the sample with its mass percent. The result shows the presence of oxygen (34.65%), silver (31.13%), iron (18.94%) and copper (15.28%). The presence of all three metals (Ag, Fe and Cu) confirms the successful synthesis of the trimetallic nanomaterial system. However, some other peaks were also observed, which might be due to the presence of some impurities from precursor salts and plant extract.

#### 2.1.3 XRD analysis

High-intensity peaks in the XRD pattern indicate the formation of Ag/Fe/Cu-TNAs. The XRD spectrum was interpreted using the High Score Plus Software (version 5.3a), which revealed the presence of cubic crystals of Ag/Fe/Cu-TNAs (Fig. 2b). Peaks appeared at 2*ϴ* = 37.8°, 44.04°, 64.04°, 76.88° and 80.9°, corresponding to the (111), (020), (022), (131), and (222) *hkl* planes, respectively, and are consistent with the conventional face-centered cubic (FCC) phase pattern of Ag-NPs (JCPDS: 96-901-3047). Fe-NPs had typical peaks corresponding to the (011), (020) and (121) planes of body-centered cubic (BCC) symmetry at 2*ϴ* values of 44.59°, 64.90° and 82.17°, respectively (JCPDS: 96-411-3942). Cu peaks overlapped with those of Ag and appeared at 2*ϴ* = 38.21° (111), 44.41° (020), 64.2° (022), 77.62° (131) and 81.788° (222), corresponding to the AgCu-NPs in accordance with JCPDS: 96-150-9855. The presence of separate and distinct phases indicates the synthesis of trimetallic nanocomposites. The crystallite size was calculated using the Scherrer Equation.^45^ The size of the crystal was found to be 17.83 nm.

#### 2.1.4 Optical properties

**Fig. 3:**
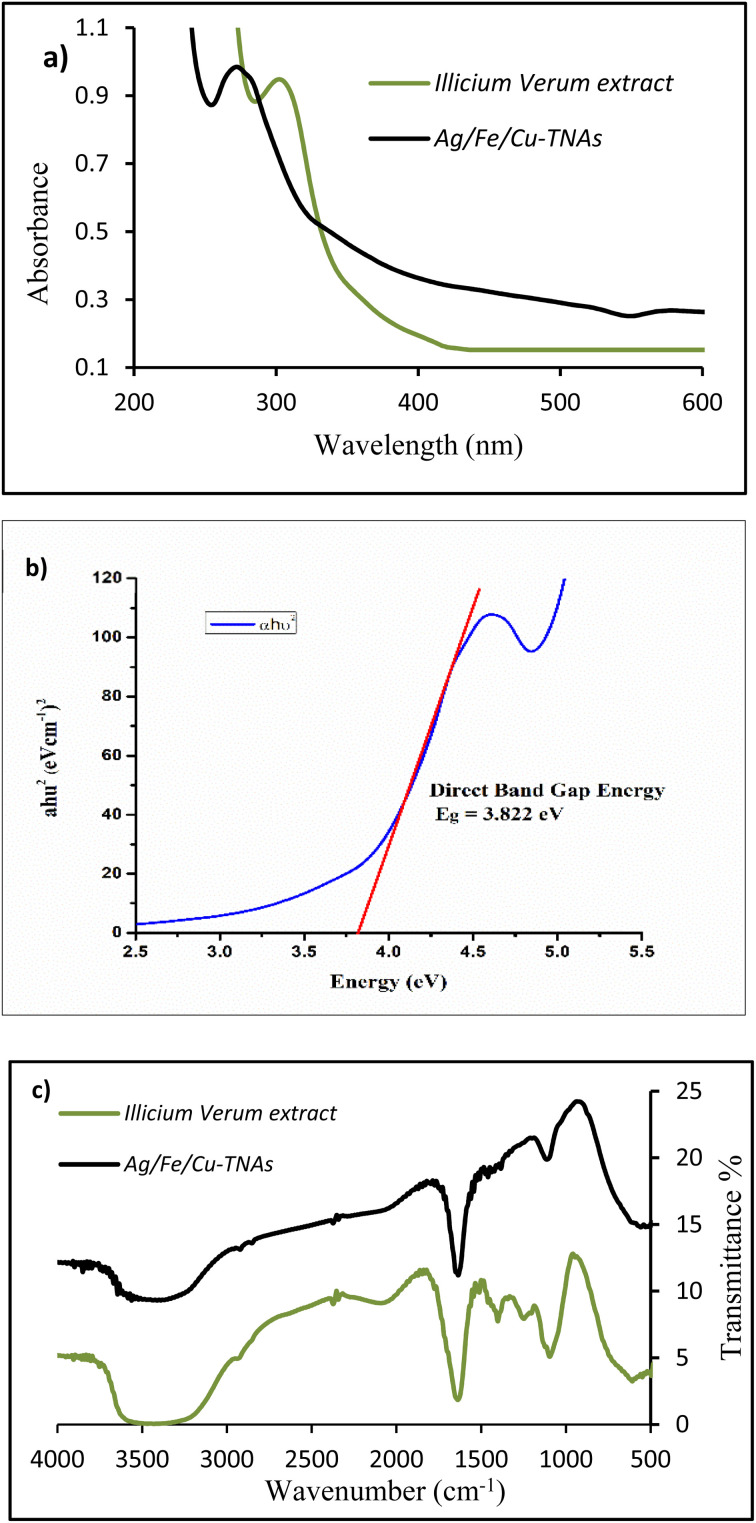
(a) UV-visible spectra, (b) Tauc plot and (c) FTIR spectra of Ag/Fe/Cu-TNAs.

The optical properties of the synthesized Ag/Fe/Cu-TNAs were determined by UV-visible spectroscopy. For the plant extract, two broad peaks appeared between 200 and 400 nm, reflecting the presence of organic chromophore groups including phenols and flavonoids, which contain π-conjugated systems.^48,49^ The peaks of CuO, AgO and FeO nanomaterials usually appear at 230–250 nm, 270–290 nm and 290–350 nm, respectively.^50^ In the UV-visible spectrum of Ag/Fe/Cu-TNAs (Fig. 3a), the broadening of the peak started at 430 nm with the spike in absorbance at 280 nm and terminated at 250 nm. This merged, broad and shifted peak indicated the successful synthesis of phytochemically stabilized trimetallic nanocomposites.

The Tauc plot is used to investigate the intrinsic optical band gap. It is mathematically represented as follows:^51^(*αE*)^1/*r*^ = *A*(*E* − *E*_g_)where *α* is the coefficient of absorption, *E*_g_ is the optical band gap energy, *A* is a constant, and *E* is the photon energy also given by *hʋ* and *r* = 1/2 for direct-band-gap transition.

The optical band gap of Ag/Fe/Cu-TNAs was computed from (*αhʋ*)^1/*r*^*versus* photon energy (eV) plot and by extending the linear tangent of the curve to intersect the abscissa (Fig. 3b).The obtained result revealed the direct transition band gap of 3.82 eV, which is consistent with the value reported in the literature and demonstrates the semiconducting nature of Ag/Fe/Cu-TNAs.^52^

#### 2.1.5 FTIR analysis

In FTIR analysis (Fig. 3c), the broad peak at ∼3600–3000 cm^−1^ corresponds to the presence of the –OH group in different compounds including phenols, flavonoids, alcohols and carboxylic acids. Strong vibration appearing at 1637 cm^−1^ is attributed to the C=O group as a part of ketone-, aldehyde- and carboxylic acid-containing compounds and 2063 cm^−1^ represents the alkyne moiety. The peaks at 1396, 1238 and 1089 cm^−1^ indicate the existence of alcoholic –OH bending and C–N stretching, respectively.^53^ In the FTIR spectra of Ag/Fe/Cu-TNAs, the increasing intensity of vibrations and the disappearance of peaks from 1000 to 1400 cm^−1^ suggested the involvement of respective functional groups in the formation of nanocomposites.

### 2.2 Screening of pharmacologically active compounds

The screening of pharmaceutical residues was carried out by nanocomposites as an effective sensing platform. Its high surface area-to-volume ratio and distinctive physicochemical properties facilitate strong interactions between the targeted molecule and the nanosensor. Measurable spectral changes are expected, which serve as a signal to evaluate the positive interaction with the analyte. This phenomenon of sensing is helpful in the determination of the presence of drug molecules in water, which cause pollution.

For this purpose, different pharmacologically active compounds, including levocetirizine, sulbactam sodium, thymine, sertraline, pyridoxine, ceftriaxone, ciprofloxacin, levofloxacin, ephedrine and cefotaxime, were screened using Ag/Fe/Cu-TNAs through UV-visible spectroscopy.

**Fig. 4:**
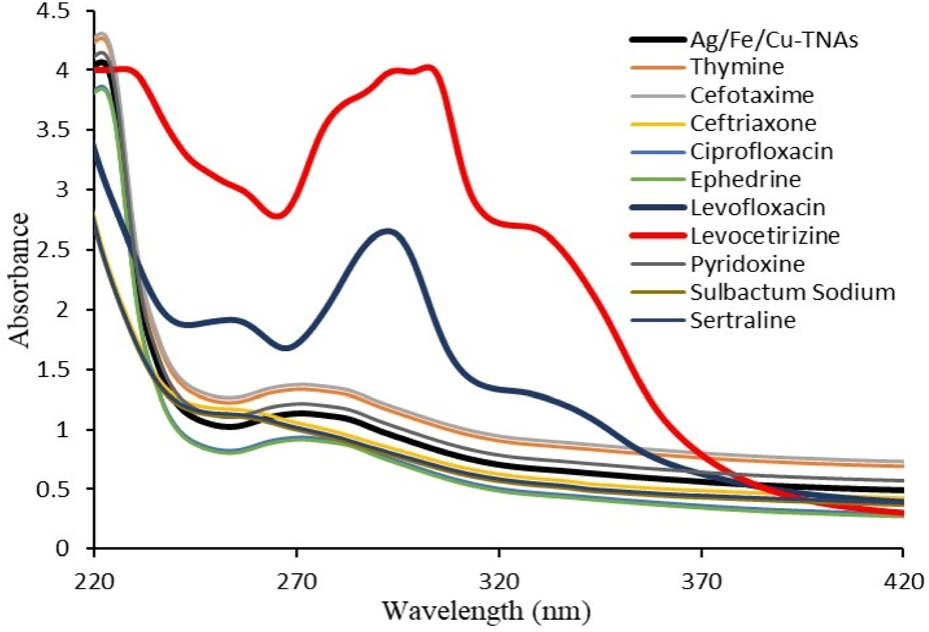
Screening of pharmaceutical residues by Ag/Fe/Cu-TNAs.

**Fig. 5:**
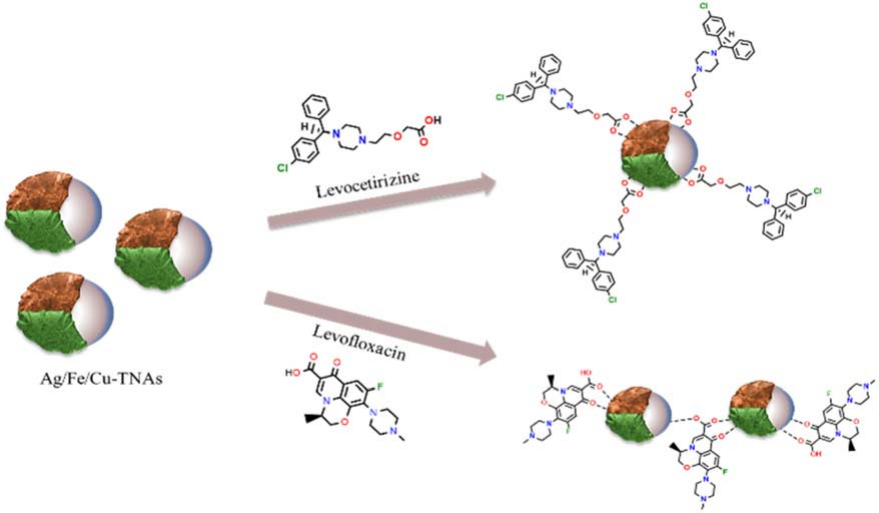
Proposed interactions between Ag/Fe/Cu-TNAs and drug molecules.

A sharp increase in intensity and bathochromic shift was detected in the case of levocetirizine and levofloxacin (Fig. 4). It reflects the strong interaction of Ag/Fe/Cu-TNAs with the two drugs (Fig. 5), while the rest of the drugs did not show any notable change in the spectra. Special intermolecular charge transfer is one of the reasons for the increasing intensity in the UV-visible spectra, while the shift in wavelength might be due to the formation of hydrogen bonding between the nanocomposites and the drug molecules.^54^ The comparative increase in the absorbance of levocetirizine as compared to levofloxacin may be due to the smaller size and strong binding capacity of the levocetirizine molecule with Ag/Fe/Cu-TNAs.^55^

### 2.3 Proposed interactions between Ag/Fe/Cu-TNAs and pharmaceutical compounds

The proposed interactions between Ag/Fe/Cu-TNAs and pharmaceutical compounds were evaluated on the basis of changes in the UV-visible and FT-IR spectra by observing the shifts in the position and intensity of peaks with the validation of previously reported data.

The electronic bands of organic molecules usually appear in the UV and visible region. The excitation of electrons from the HOMO to the LUMO results in the transition of molecules to a higher electronic state. The transition band at a wavelength less than 200 nm often occurs due to the transition of electrons from sigma (bonding) to sigma (antibonding) molecular orbital. In contrast, the bands near the regions of ultraviolet and visible regions appeared due to the π – π* and n – π* transitions while the n – σ* transition was observed at a longer wavelength.^56^ In the UV-visible spectra of levocetirizine (Fig. S1, SI), the weak band at 315 nm is due to the charge transfer from the nucleophilic carboxylate group to the electrophilic piperazine ring. At 291 nm, a strong band appeared due to the π – π* transition between two benzene rings.^57^ In levofloxacin (Fig. S2, SI), the peaks at 343, 292 and 260 nm in the UV – visible spectra are attributed to the π – π* and n – π* transitions.^58^ After the association of drug molecules with nanocomposites, the broadening, increasing intensity and bathochromic (red) shift of the peak by ∼13 nm (291–304 nm) and ∼16 nm (292–308 nm), respectively, indicated the strong interaction between drug molecules and nanocomposites. The shift in lower energy might be due to the stabilization of the π* orbital of drug molecules after coordination with nanocomposites.^59^

In the IR spectra of levocetirizine (Fig. S3, SI), the peak at 1724 cm^−1^ corresponds to the carbonyl group of carboxylic acid and the band around 1602 cm^−1^ represents the aromatic ring. The bands at 1479, 1319 and 1283 cm^−1^ appear due to the presence of a C–O bond in carboxylic acid, C–N stretching of the piperazine ring and C–O bond in ether, respectively, while the peak at 875 cm^−1^ is attributed to C–Cl stretching.^60^ In a recent study by Salem *et al.*, it was shown that levocetirizine acts as a monodentate ligand through its carboxylate oxygen atom.^61^ The shift of C=O peak to 1685 cm^−1^ represented the involvement of a carboxylate group in the association.^57^ In the IR spectra of levofloxacin (Fig. S4, SI), the peaks at 1724 and 1620 cm^−1^ could be assigned to the carbonyl functionality present in carboxylic acid and ketone, respectively. The O–H stretching vibration of carboxylic acid and the N–H stretching vibration of the piperazinyl moiety were observed between 3265 and 3500 cm^−1^. The peak at 937 cm^−1^ is ascribed to the C–F bond, while the bands of aromatic ring are present between 1415 and 1509 cm^−1^.^62^ Previous studies showed that levofloxacin binds as a bidentate ligand through the oxygen atoms of its carboxyl and pyridone carbonyl groups. The shifting in the absorption band for the stretching vibration of carbonyl groups to 1649 cm^−1^ and 1627 cm^−1^ is an strong evidence for the participation of the following groups in association with nanocomposites.^58^

### 2.4 Determination of the stoichiometric ratio and binding constant

**Fig. 6:**
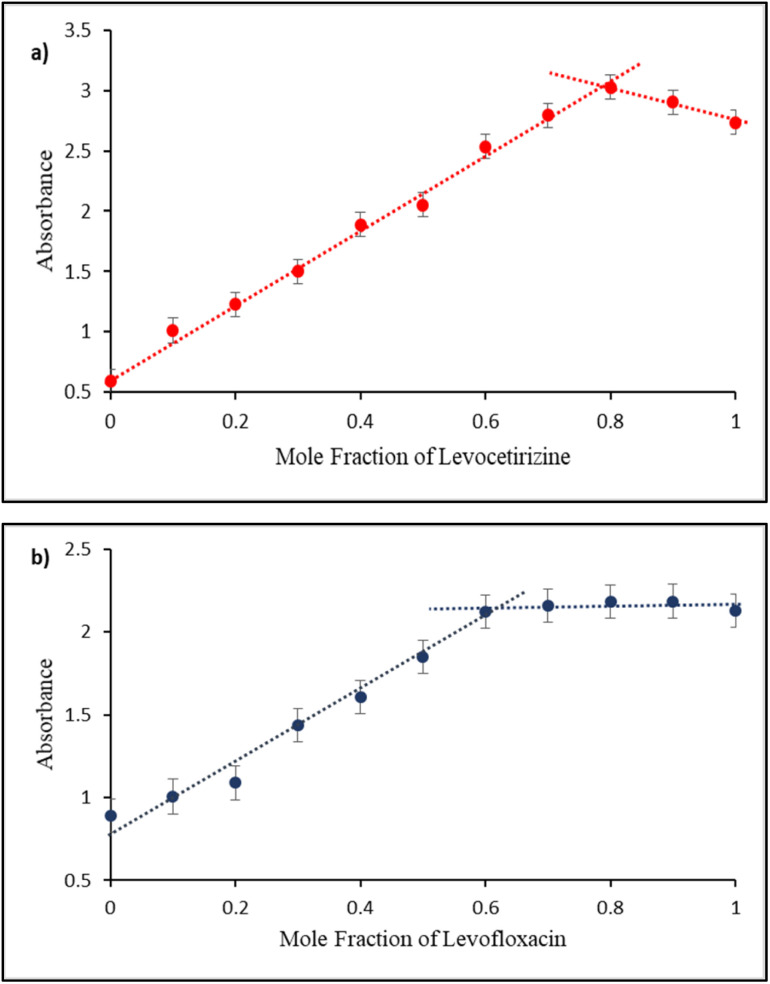
Job's plot for Ag/Fe/Cu-TNAs with (a) levocetirizine and (b) levofloxacin.

The stoichiometric ratio was determined from Job's plot which revealed that Ag/Fe/Cu-TNAs interact with levocetirizine in a ratio of 1 : 4 (Fig. 6a), while the ratio between Ag/Fe/Cu-TNAs and levofloxacin was found to be 2 : 3 (Fig. 6b).

Absorption titration data were used to determine the binding constant (*K*_a_) for Ag/Fe/Cu-TNAs with drug molecules. Its value was calculated using the Benesi–Hildebrand equation:
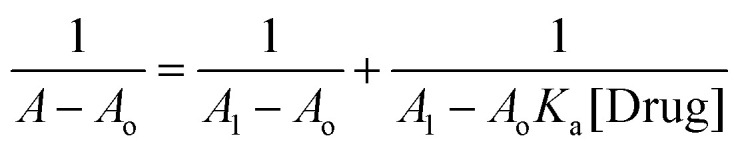
where *A*_o_ is the absorbance of Ag/Fe/Cu-TNAs, *A* is the absorbance in the presence of drugs, and *A*_1_ is the absorbance upon maximum binding with drug molecules.

The *K*_a_ value for levofloxacin and levocetirizine with nanocomposites was found to be 1.139 × 10^4^ M^−1^ (Fig. S5, SI) and 1.153 × 10^5^ M^−1^ (Fig. S6, SI), respectively. These values suggest that Ag/Fe/Cu-TNAs are strongly bound with levocetirizine as compared to levofloxacin.^63^

### 2.5 Effect of temperature on Ag/Fe/Cu-TNAs and its sensing ability

**Fig. 7:**
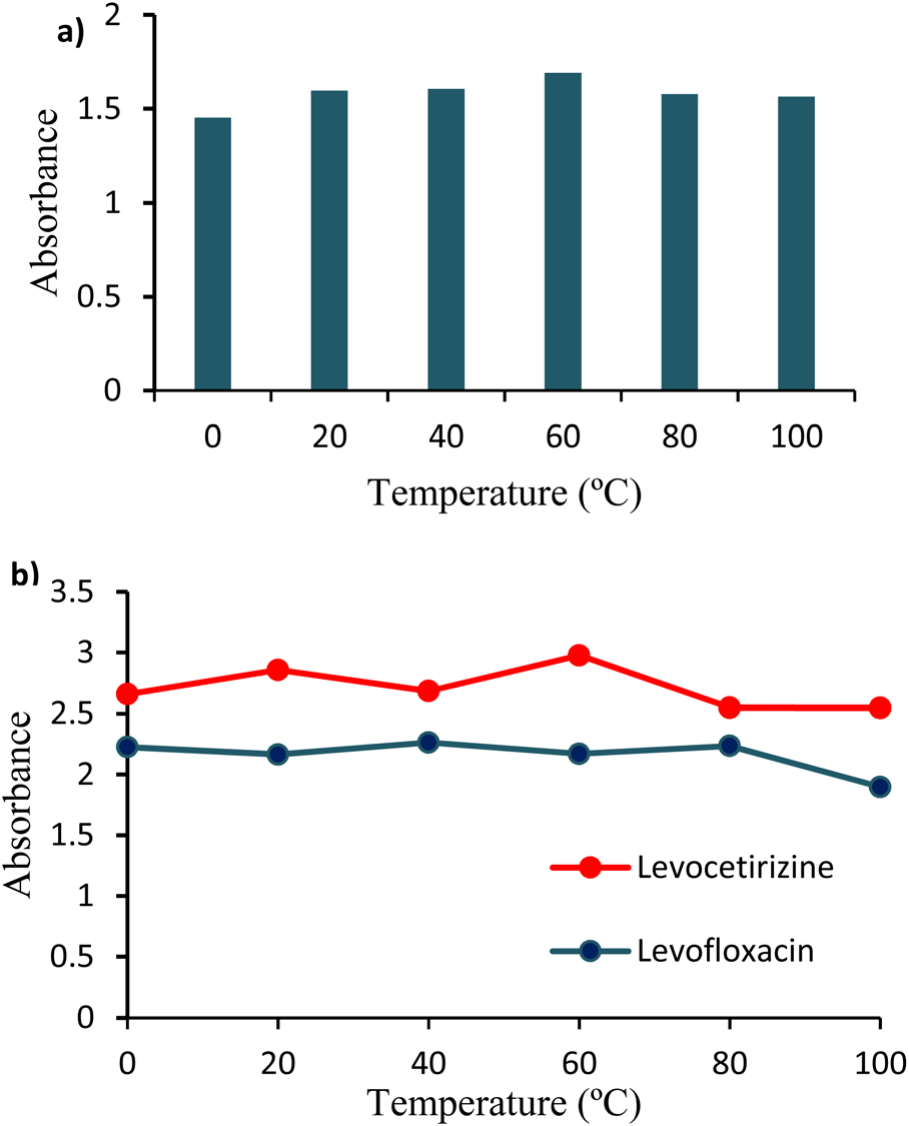
Effect of temperature on (a) Ag/Fe/Cu-TNAs and (b) its interaction with drug molecules.

The absorbance of Ag/Fe/Cu-TNAs and its association with drug molecules were measured over a range of temperatures (0–100 °C). No significant change in the absorbance of Ag/Fe/Cu-TNAs was observed, which revealed their stability over a wide range of temperatures (Fig. 7a). A similar pattern was demonstrated for the interactions of Ag/Fe/Cu-TNAs with both drugs. The absence of any noticeable change in the absorbance reflects that the associations are highly stable, and hence, the affinity of drug molecules with Ag/Fe/Cu-TNAs is not affected by temperature (Fig. 7b).

### 2.6 Effect of pH on Ag/Fe/Cu-TNAs and its sensing ability

**Fig. 8:**
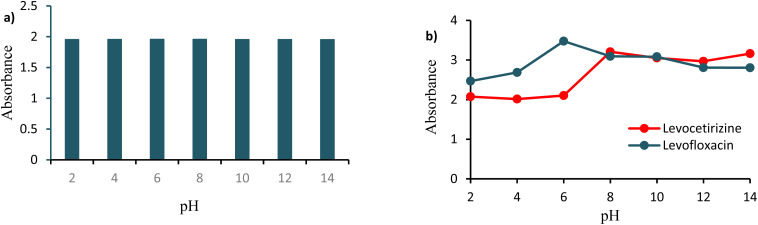
Effect of pH on (a) Ag/Fe/Cu-TNAs and (b) their interactions with drug molecules.

The effect of pH on the stability was determined by taking absorbance at various pH values (2–14) maintained by using HCl and NaOH solutions. As shown in Fig. 8a, the nanocomposites were found to be stable over an expanded range of pH.

In Fig. 8b, the increase in the absorption of the levofloxacin-Ag/Fe/Cu-TNA assembly at acidic pH is due the formation of strong bonding as levofloxacin is more ionized and soluble in water at a pH around 3–6. In a basic medium, the absorbance of the levofloxacin-Ag/Fe/Cu-TNA conjugate was decreased due to deionization and lower solubility.^64^ In the case of levocetirizine and Ag/Fe/Cu-TNAs, the decrease in absorbance in acidic media may be ascribed to acidic hydrolysis. However, it remained stable in a basic medium as the basic condition had no effect on the stability of levocetirizine.^65^

### 2.7 Selectivity analysis of Ag/Fe/Cu-TNAs

**Fig. 9:**
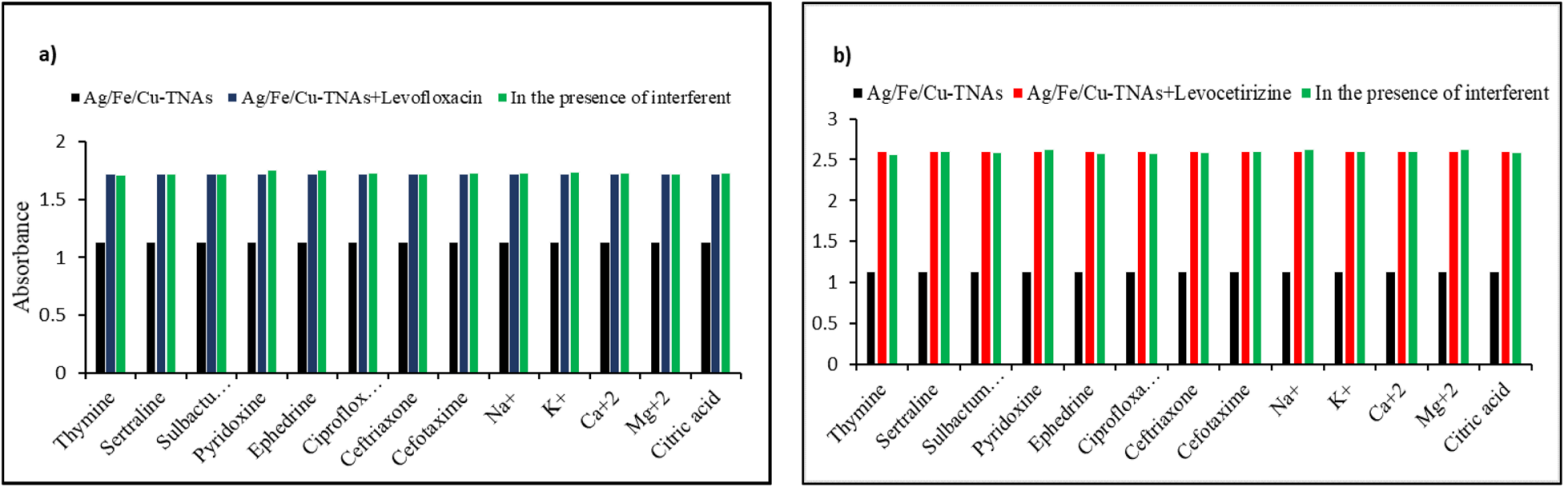
Selectivity analysis of Ag/Fe/Cu-TNAs for (a) levofloxacin and (b) levocetirizine.

The selectivity of Ag/Fe/Cu-TNAs for levofloxacin and levocetirizine was identified by interference analysis. In this study, the absorbance of solutions containing equal volumes of levofloxacin and Ag/Fe/Cu-TNAs, along with other drugs, was measured at 295 nm. The experimental evidence indicated that levofloxacin exhibited a remarkable affinity with Ag/Fe/Cu-TNAs (Fig. 9a).

Meanwhile, the interaction between levocetirizine and Ag/Fe/Cu-TNAs was evaluated in the presence of other drugs in equal volumes. The lack of any significant change in absorbance at 294 nm suggests high selectivity of Ag/Fe/Cu-TNAs towards levocetirizine (Fig. 9b). In addition, the effect of different ions including Na^+^, K^+^, Ca^2+^, Mg^2+^ and citric acid was also studied to validate the selectivity of the nanosensor. The results showed the prominent efficiency of nanosensors even in complexed media.

### 2.8 Analytical sensitivity determination of Ag/Fe/Cu-TNAs

**Fig. 10:**
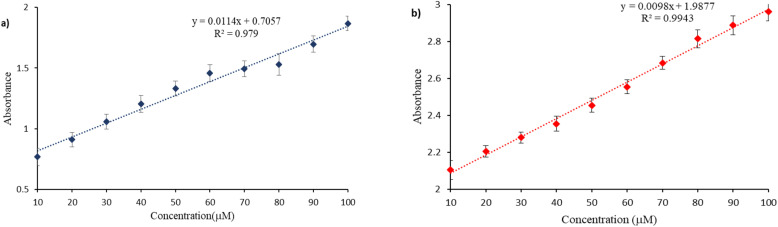
Calibration curves of (a) levofloxacin and (b) levocetirizine.

The analytical sensitivity of Ag/Fe/Cu-TNAs was determined by estimating the limit of detection (LOD) and limit of quantification (LOQ) for levofloxacin and levocetirizine. It was evaluated by analyzing absorbance at decreasing concentrations of both drugs (Fig. S7 and S8, SI). Linear interval methodology was applied in which the absorbance is correlated with the decrease in the concentration of drugs, which is supported by regression values. Data were obtained in replicate and are presented as mean ± S.D (standard deviation). The LOD and LOQ were calculated using the following formula:
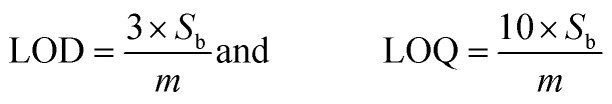
where *s* is the standard deviation (blank) and *m* is the sensitivity (determined by the slope of calibration curve).^66^ The formula revealed the LOD and LOQ values of 4.43 µM and 13.42 µM for levofloxacin (*R*^2^ = 0.98) and 5.494 µM and 16.649 µM for levocetirizine (*R*^2^ = 0.99), respectively (Fig. 10a and b).

### 2.9 Detection of drugs in tap and well water

To further examine the effectiveness of Ag/Fe/Cu-TNAs as biosensors, levofloxacin and levocetirizine were spiked in tap and well water. Ag/Fe/Cu-TNAs were found to be effective sensors for these drugs in real samples, as the UV-visible spectra represented the same results obtained in deionized water (Fig. S9–S12, SI). The selectivity of nanosensors in complex media was also evaluated by spiking other drugs in the solution of nanocomposites and respective analytes. The results (Fig. S13–S16, SI) also illustrated the excellent selectivity in real water.

The minimal dosage of levocetirizine is 5 mg once daily,^67^ and the therapeutic levels for levofloxacin are generally between 0.10 and 5.00 mg L^−1^ (ref 33) depending upon the infection type, which has to be treated. The study stated an LOD value of 4.43 µM for levofloxacin and 5.494 µM for levocetirizine. These LOD values are in good agreement with the allowed minimal dosage for these drugs, suggesting significant sensitivity for clinical examinations and safer sensors, as Ag/Fe/Cu-TNAs are synthesized by a green method.

### 2.10 Optimization of the dye degradation process

**Fig. 11:**
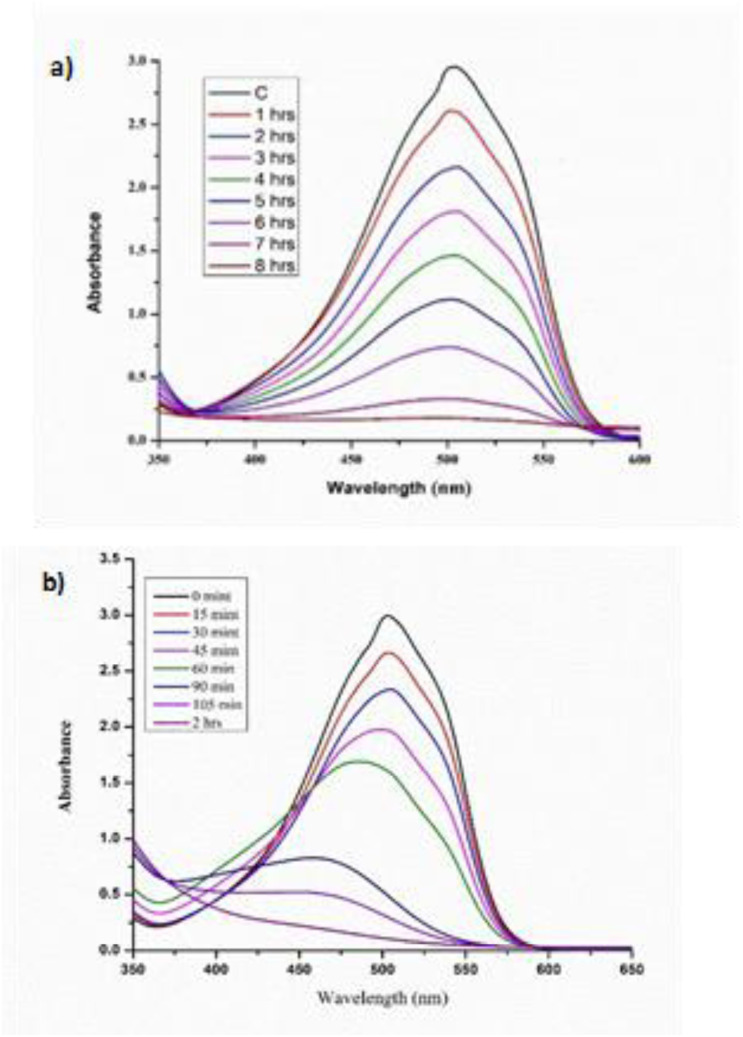
Degradation of methyl orange with H_2_O_2_ (a) in the absence of Ag/Fe/Cu-TNAs and (b) in the presence of Ag/Fe/Cu-TNAs.

Ag/Fe/Cu-TNAs were evaluated for their catalytic efficiency in H_2_O_2_-mediated degradation of methyl orange. Control experiments were conducted to verify the catalytic contribution of Ag/Fe/Cu-TNAs. The degradation of methyl orange was examined under two different conditions: (a) H_2_O_2_ only and (b) Ag/Fe/Cu-TNAs with H_2_O_2_. Negligible degradation was observed in the absence of the nanocatalyst, whereas the combined system exhibited rapid dye removal, confirming the synergistic effect required for Fenton-like activity. Degradation was carried out *via* a heterogeneous Fenton-like mechanism.^68^ The absorbance spectra were recorded progressively until a minimum absorbance was achieved (Fig. 11a and b).

### 2.11 FTIR analysis of methyl orange degradation

**Fig. 12:**
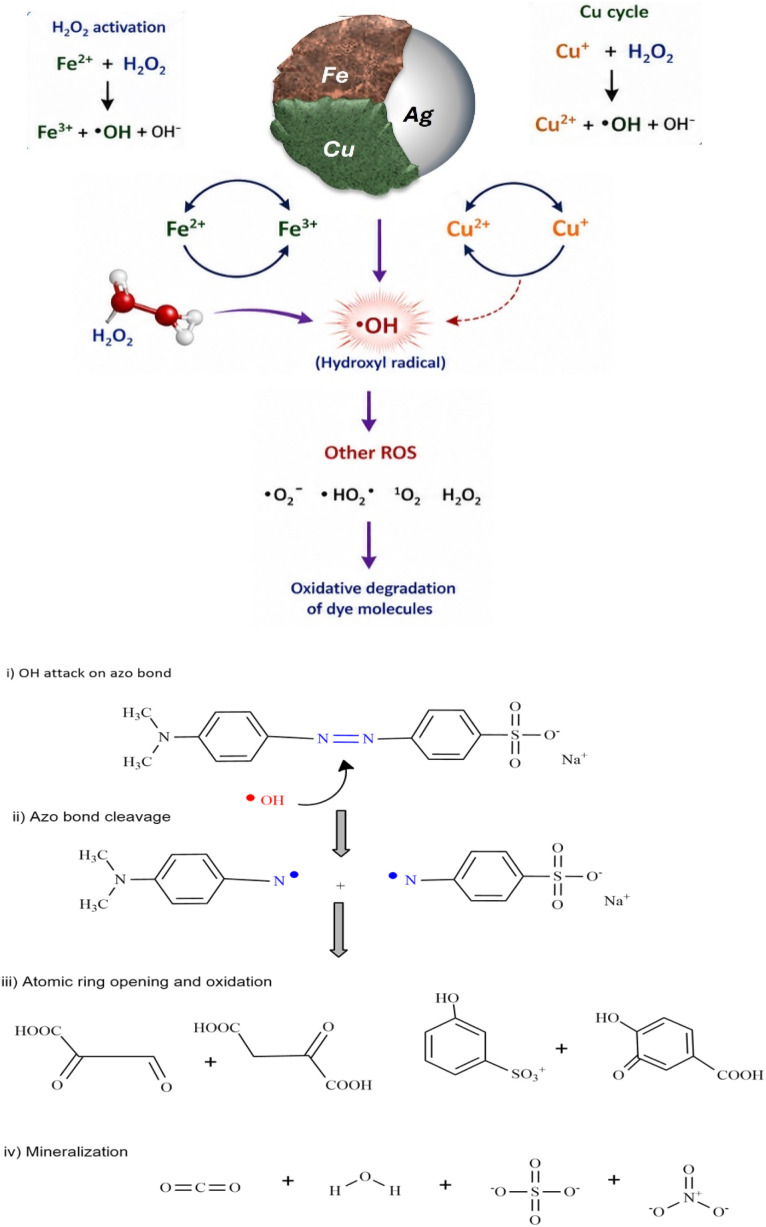
Proposed mechanism of the Fenton-like degradation of methyl orange in the presence of Ag/Fe/Cu-TNAs.

The structural differences in methyl orange (MO) before and after catalytic degradation utilizing Ag/Fe/Cu trimetallic nanocomposites (TNAs) were analyzed using Fourier transform infrared (FTIR) spectroscopy. The spectral features are illustrated in Fig. 12.

The FTIR spectrum of MO (Fig. S17, SI) before degradation exhibits characteristic absorption bands at ∼1600 cm^−1^, assigned to the azo (–N=N–) linkage. A significant reduction in intensity was noted after catalytic treatment, indicating the cleavage of the chromophore (–N=N–) bond,^69^ along with aromatic C=C stretching vibrations. Simultaneously, the weakening of aromatic ring vibrations (1500–1450 cm^−1^) and C–H bending modes (900–700 cm^−1^) suggests the disruption of the benzene framework. The decrease in the intensity of sulfonate-related bands (1190–1120 and ∼1030 cm^−1^) further confirms the desulfonation and removal of substituent groups during the degradation process. In contrast, the O–H stretching region (3400–3200 cm^−1^) becomes broader and more intense after degradation, indicating the formation of hydroxylated and oxidized intermediates.^70^

These observations provide clear evidence that the removal of methyl orange is not limited to decolorization but involves substantial chemical transformation. The spectral changes are consistent with the proposed Fenton-like mechanism, in which hydroxyl radicals (^•^OH) generated from H_2_O_2_ activation attack the azo bond, followed by the progressive oxidation of aromatic intermediates and eventual mineralization.

### 2.12 Fenton-like mechanism of dye degradation

The catalytic degradation of methyl orange (MO) in the presence of Ag/Fe/Cu-TNAs and H_2_O_2_ is proposed in Fig. 12 to proceed *via* a Fenton-like mechanism. In such systems, transition metal ions (particularly Fe^2+^/Fe^3+^ redox pairs) activate hydrogen peroxide to generate highly reactive oxygen species (ROS), which are responsible for the oxidative degradation of organic pollutants.

The primary step involves the activation of H_2_O_2_ on the catalyst surface:^71^Fe^2+^ + H_2_O_2_ → Fe^3+^ + ˙OH + OH^−^

The generated hydroxyl radicals (˙OH) are strong, non-selective oxidants capable of rapidly attacking the azo bond (–N=N–) and aromatic rings of methyl orange, leading to fragmentation and eventual mineralization into smaller, less harmful molecules such as CO_2_ and H_2_O.^72^

The presence of Cu and Ag in the trimetallic system further enhances the catalytic efficiency through synergistic effects.^73^ Copper ions (Cu^+^/Cu^2+^) can also participate in Fenton-like reactions, promoting additional ROS generation as follows:Cu^+^ + H_2_O_2_ → Cu^2+^ + ˙OH + OH^−^

Meanwhile, Ag nanoparticles improve the electron transfer processes and reduce the electron–hole recombination, facilitating continuous regeneration of active metal species. This synergistic interaction among Fe, Cu, and Ag results in enhanced production of reactive species and improved degradation efficiency.

### 2.13 Percent removal of methyl orange in the presence and absence of Ag/Fe/Cu-TNAs

**Fig. 13:**
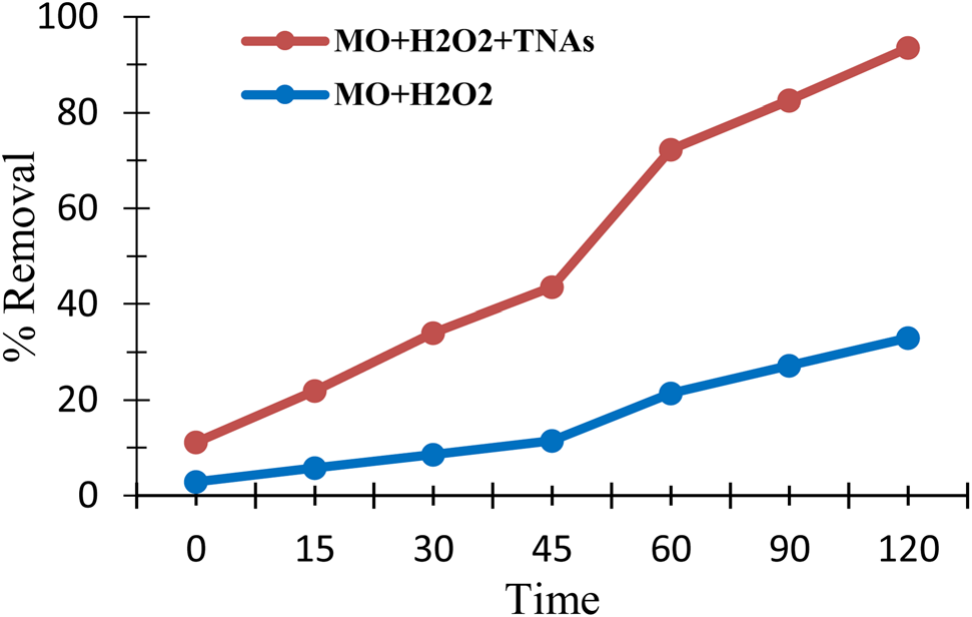
Percent removal of methyl orange in the presence and absence of Ag/Fe/Cu-TNAs.

The elimination of methyl orange dye by using nanocomposites/H_2_O_2_ system was successfully achieved in 2 hours with 93.48% elimination efficiency (Fig. 13), in contrast to H_2_O_2_ alone with 33.00%, which attained the results in 8 hours under neutral conditions and room temperature. To exclude the pH-driven effects, experiments were conducted under comparable pH conditions, ensuring that the observed degradation is primarily due to the catalytic activity rather than pH variation.

### 2.14 Kinetic study of methyl orange degradation in the presence and absence of Ag/Fe/Cu-TNAs

In heterogeneous catalytic systems involving dye degradation, the reaction mechanism is often described by the Langmuir–Hinshelwood (L–H) model, which considers both adsorption of the reactant on the catalyst surface and subsequent surface reaction. The general L–H rate expression is given as follows:
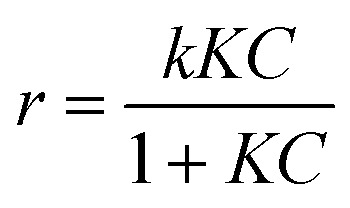
where *k* is the intrinsic surface reaction rate constant, *K* is the adsorption equilibrium constant, and *C* is the concentration of the dye.

Under dilute conditions (*i.e.*, when *KC* ≪ 1), the denominator approaches unity, and the L–H model simplifies to a pseudo-first-order kinetic expression.^74^*r* = *k*_app_*C*where *k*_app_ = *kK* is the apparent rate constant. Upon integration, this yields:ln(*C*/*C*_*o*_) = −*k*_app_*t*where *C*_0_is the initial dye concentration and *C* is the concentration at time *t*.

Since the degradation experiments were conducted at relatively low dye concentrations, the linear relationship observed in ln(*C*/*C*_0_) *vs.* time confirms the pseudo-first-order kinetics derived from the L–H mechanism under dilute conditions. The high linear correlation coefficient (*R*^2^ = 0.9117) further supports the validity of this kinetic model.^75^ln *C*/*C*_o_ = −*K* × *t*

The results obtained from dye degradation in the presence and absence of Ag/Fe/Cu-TNAs were analyzed by plotting the normalized concentration (*C*/*C*_0_) and its logarithmic form (ln *C*/*C*_0_) as a function of time. All degradation experiments were conducted in triplicate to ensure reproducibility. The apparent rate constant (*k*_app_) was calculated from the slope (0.01875) of the linear fit of ln(*C*/*C*_0_) *versus* time. The values are reported as mean ± standard deviation.

**Fig. 14:**
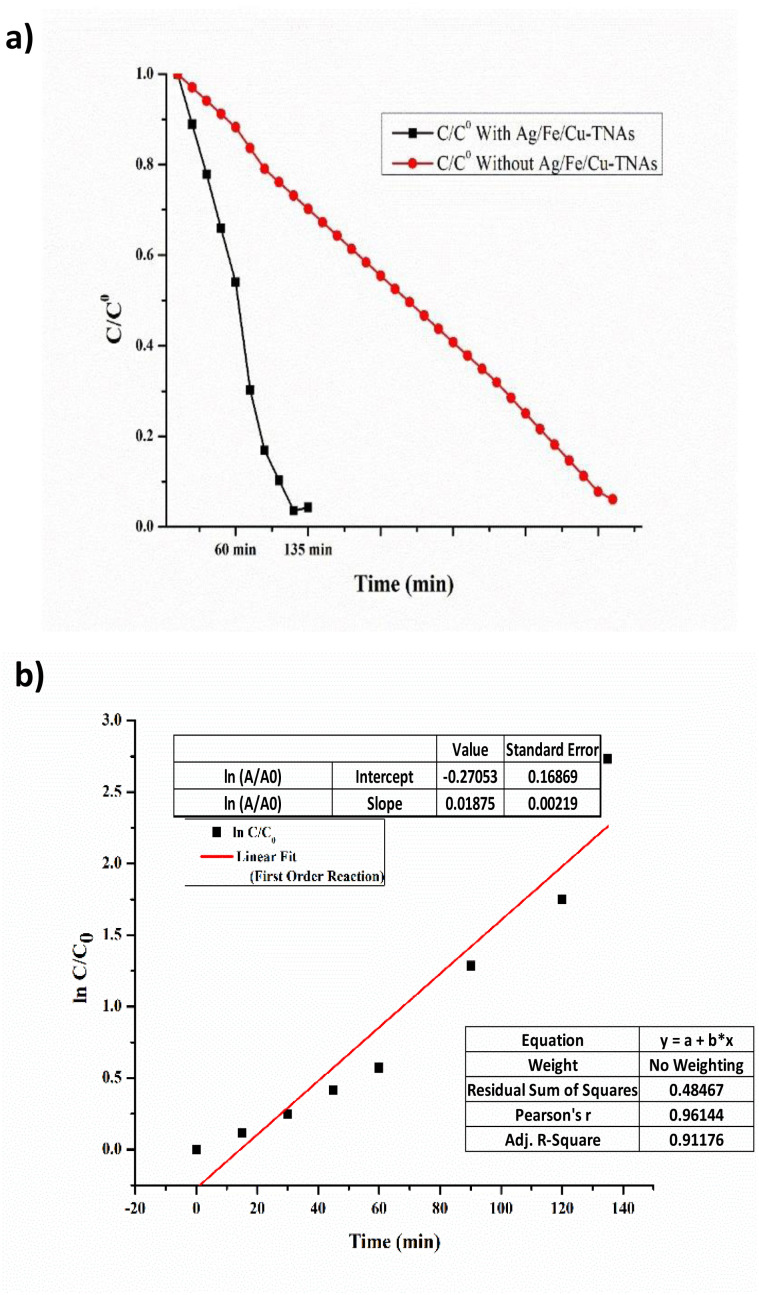
First-order kinetics graph plots of (a) normalized C/Co and (b) logarithmic form (ln *C*/*C*_o_) *versus* time for the catalytic degradation of methyl orange.

As shown in Fig. 14a, the methyl orange concentration declines instantly in the presence of Ag/Fe/Cu-TNAs as compared to bare H_2_O_2_. It shows that the removal efficiency was considerably enhanced by using these nanocomposites. The enhanced performance can be attributed to the increased surface adsorption of dye molecules and improved generation of reactive species on the catalyst surface. The steady decline pattern of ln *C*/*C*_o_*vs.* time (Fig. 14b) explained the catalytic decay of methyl orange and also supported a first-order reaction, which is consistent with the Langmuir–Hinshelwood mechanism under low-concentration conditions. Pearson's *r* value of 0.9614 also indicates a significant linear relationship between time and concentration.

## 3. Experimental

### 3.1 Instrumentation

A Shimadzu UV-240 spectrophotometer was used to record the UV-visible spectra in the range of 200–800 nm, utilizing a quartz cell of 1 cm path length. A PHS-3B microprocessor pH meter was used to measure the pH. FTIR spectroscopy was performed using a Shimadzu IR-Prestige-21 with KBr pellets. The size and shape of Ag/Fe/Cu-TNAs were estimated by scanning electron microscopy (SEM) and atomic force microscopy (AFM) using a JEOL JSM-6380A (Japan), a JFC-1500 sample coater and an Agilent Technologies 5500 microscope (USA). The elemental compositions of Ag/Fe/Cu-TNAs were obtained through energy-dispersive X-ray spectroscopy (sample was coated up to 300 Å with gold, Model # EX-54175IMU, JEOL Japan). The crystallinity and phase were determined by XRD analysis using a Panalytical X'Pert Pro.

### 3.2 Materials and methods

*Illicium verum* fruits (Badyan) were purchased from online herbal mart (Karachi Pansar), Pakistan. Salts (FeSO_4_, Cu(CH_3_COO)_2_ and AgNO_3_), methyl orange dye and drugs (sertraline, pyridoxine, cefotaxime, ceftriaxone, ciprofloxacin, levocetirizine, levofloxacin, sulbactam sodium, ephedrine and thymine) were purchased from Sigma-Aldrich.

### 3.3 Extract preparation

**Fig. 15:**
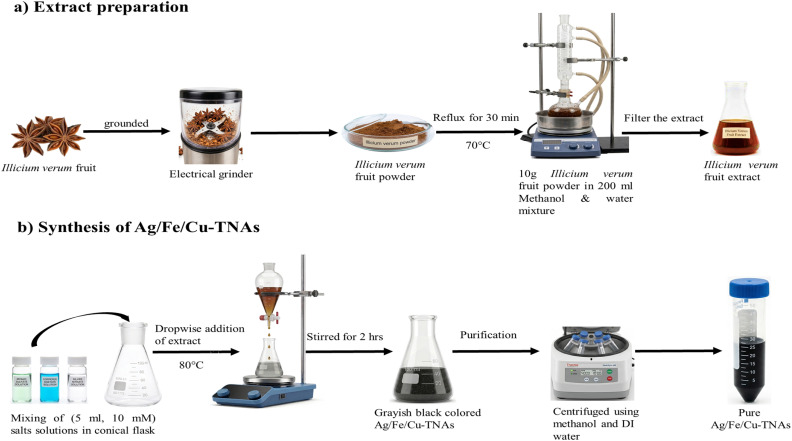
Schematic of the (a) extract preparation and (b) synthesis of Ag/Fe/Cu-TNAs.

Fruits of *Illicium verum* were washed with deionized water and air-dried for 24 hours. The dried fruits were grounded using an electrical grinder. Then, 10 g powder was taken into a 500 mL round-bottom flask containing 200 mL methanol : deionized water (1 : 1). The contents of the flask were refluxed at 70 °C for 30 min. The extract was collected using vacuum filtration and used for the synthesis of Ag/Fe/Cu-TNAs (Fig. 15).

### 3.4 Green synthesis of Ag/Fe/Cu-TNAs

Ag/Fe/Cu-TNAs were synthesized by the bio reduction of precursor salts with the fruit extract. 5 mL solution (10 mM) of each salt [FeSO_4_·7H_2_O, Cu(AcO)_2_·H_2_O and AgNO_3_] was mixed with each other in a 100 mL Erlenmeyer flask and stirred for 15 minutes. The *Illicium verum* extract (15 mL) was added dropwise into the salt solution with 5 minute intervals at 80 °C. The reaction proceeded at the intrinsic pH of plant extract. The formation of greyish-green color precipitates indicated the formation of Ag/Fe/Cu-TNAs (Fig. S18, SI). The reaction was continued for two hours with constant stirring for optimization. The collection of nanocomposites was carried out by centrifugation, and washing was done by using deionized water followed by methanol. Following purification, they were re-suspended in deionized water to facilitate extended analysis.

### 3.5 Preparation of pharmacologically active compound solutions

Working solutions (100 µM) of ten different drugs were prepared by diluting the stock solutions (1 mM), which were formulated by accurately weighing the required amount of each drug. The chemosensing potential of Ag/Fe/Cu-TNAs was evaluated by observing the UV-visible spectra of 1 : 1 mixture of trimetallic nanocomposites and drug solutions.

### 3.6 Preparation of real samples

Tap (pH = 6.6; TDS = 202 mg L^−1^) and well water (pH = 6.2; TDS = 500 mg L^−1^) were collected as a source of real sample. Solutions (100 µM) of both drugs were prepared again by using real-water samples and analyzed for comparison. Two solutions (A and B) were prepared for the analysis. Solution A contains 1 mL Ag/Fe/Cu-TNAs and 1 mL of water sample, while solution B contains 1 mL Ag/Fe/Cu-TNAs and 1 mL drug solution prepared in real-water sample. All the real sample solutions were analyzed using UV-visible spectroscopy.

### 3.7 Sensing of pharmaceutical pollutants with different analytical studies

The screening of drug molecules was performed by mixing the nanocomposite solution with a 100 micromolar solution of each drug and analyzing them *via* UV-visible spectroscopy.

The 100 micromolar solution of drugs sensed by nanocomposites was prepared later in a 100 mL volumetric flask to determine the selectivity, stability and sensitivity. The selectivity analysis was performed by spiking different drugs, Na^+^, K^+^, Ca^2+^, Mg^2+^ ions and citric acid in the mixture of nanocomposites and analyte molecules.

For stability analysis, solutions of nanocomposites and drugs at different pH values were prepared. The pH of the solutions was monitored using a pH meter. The temperature of solutions was controlled using a hot plate and a refrigerator and monitored with a thermometer.

Different solutions of nanocomposites with decreasing concentrations of drugs were prepared and analyzed to determine the limit of detection and limit of quantification of the sensor, while the solutions with different molar ratios of nanocomposites and drugs were prepared to determine the stoichiometric ratios. All the analyses were performed by UV-visible spectroscopy.

### 3.8 Preparation of methyl orange and hydrogen peroxide solutions

A methyl orange solution (5 µM) was prepared in deionized water, while a 10% H_2_O_2_ solution was obtained by the dilution of 30% industrial grade H_2_O_2_ stock solution.

### 3.9 Catalytic degradation of methyl orange by Ag/Fe/Cu-TNAs

Two solutions A and B were prepared to determine the catalytic degradation. Solution A contained methyl orange and H_2_O_2_ (2 mL each), while solution B contained 5 mg of Ag/Fe/Cu-TNAs along with the same composition as solution A. Both solutions were continuously stirred at room temperature while recording the UV-visible spectra every 15 minutes.

The percent removal of methyl orange dye was calculated using the following equation:
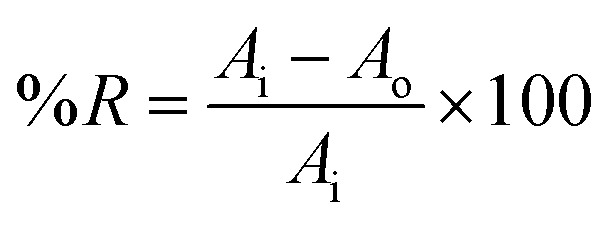
where *A*_i_ and *A*_o_ are the initial and final absorbance values of methyl orange solutions, respectively.^76^

## 4. Conclusion

In this study, silver-, iron- and copper-based semiconducting tri-metallic nanocomposites (Ag/Fe/Cu-TNAs) have been successfully synthesized from an aqueous extract of *Illicium verum* fruit. These nanocomposites were characterized by SEM, AFM, EDS and XRD techniques, while the optical properties were obtained by UV-visible spectroscopy. The XRD pattern revealed the presence of distinct phases and hence illustrated the composite material rather than interparticle mixing. The synthesized trimetallic nanocomposites served as the first ever nanosensor for the selective detection of levocetirizine. In addition to levocetirizine, they also detected levofloxacin. The impressive selectivity for levofloxacin and levocetirizine was also demonstrated even in the presence of different drug molecules. Significantly lower LOD and LOQ values for the drugs made the synthesized nanocomposites highly sensitive nanosensors, which have the potential to detect the respective molecules in a complex medium. These nanocomposites have also shown remarkable chemosensing potential for the drugs in tap and well water with prominent selectivity. Additionally, the incorporation of nanocomposites significantly accelerated dye removal and reduced the total degradation time in the absence of light. In the future, they could be employed as green, economically viable and ecologically safe semiconducting materials to reduce water pollution along with other potential applications.

## Author contributions

AZ, ZM: literature review, experimental work, data collection and writing the original draft. SFB, IM: data analysis and writing the original draft. SNA: conceptualization, reviewing, writing and editing the original draft. All authors reviewed and approved the final version of the manuscript.

## Conflicts of interest

There are no conflicts to declare.

## Supplementary Material

RA-016-D6RA02869A-s001

## Data Availability

The supporting data has been provided as the part of supplementary information (SI). Supplementary information is available. See DOI: https://doi.org/10.1039/d6ra02869a.
