# Supplementary material for: Green synthesis of Illicium verum-derived novel semiconducting Ag/Fe/Cu-trimetallic nanocomposites: A dual-functional platform for selective detection of pharmaceutical compounds and light-independent degradation of organic dyes
Source: RSC Adv. 2026 May 18;16(29):26572–84. doi: 10.1039/d6ra02869a (PMC13185807; doi:10.1039/d6ra02869a)
Supplement: RA-016-D6RA02869A-s001 [file RA-016-D6RA02869A-s001.pdf]

Supplementary Information (SI) for RSC Advances.
This journal is © The Royal Society of Chemistry 2026

Green Synthesis of *Illicium verum* derived novel semiconducting Ag/Fe/Cu-trimetallic nanocomposites: Dual functional platform for selective detection of pharmaceutical compounds and light independent degradation of organic dye

Ambreen Zia,[a] Ziana Manzar,[a] Syed Nawazish Ali,*[a,b] Syeda Farah Bukhari,[a] and Imran Malik[c]

[a]*Department of Chemistry, University of Karachi, Karachi-75270, Pakistan*

[b]*Department of Chemistry, NED University of Engineering and Technology, Karachi-75270, Pakistan*

[c]*Department of Basic Science (Chemistry), Imam Abdulrahman Bin Faisal University, Dammam, Saudi Arabia*

* syed.nawazish@gmail.com; snali@uok.edu.pk

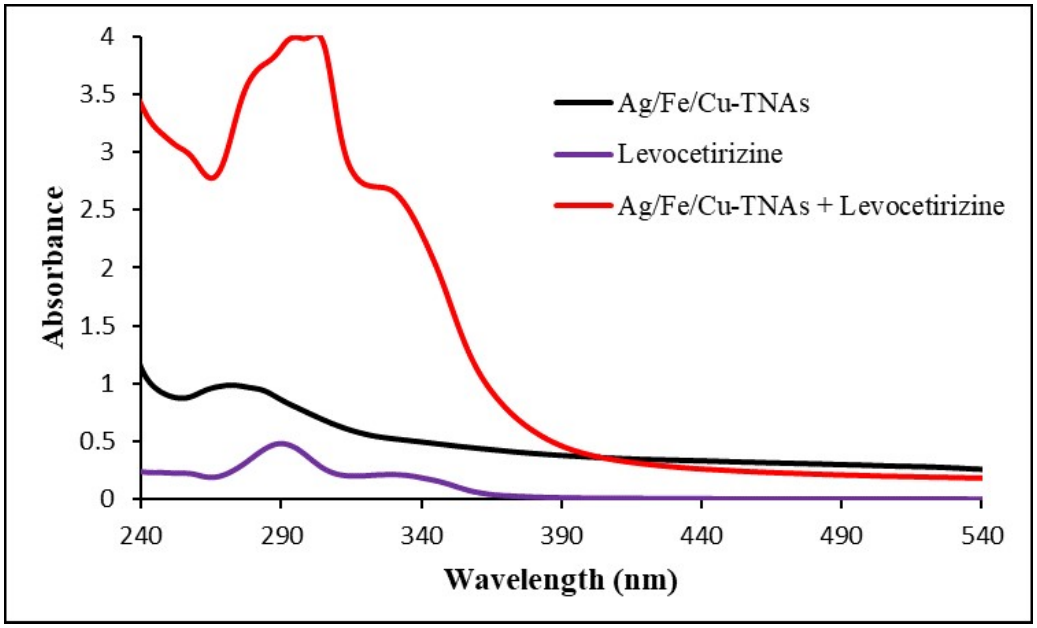

Fig. S1 Comparative UV-Visible spectra of Ag/Fe/Cu-TNAs, Levocetirizine and interaction between them

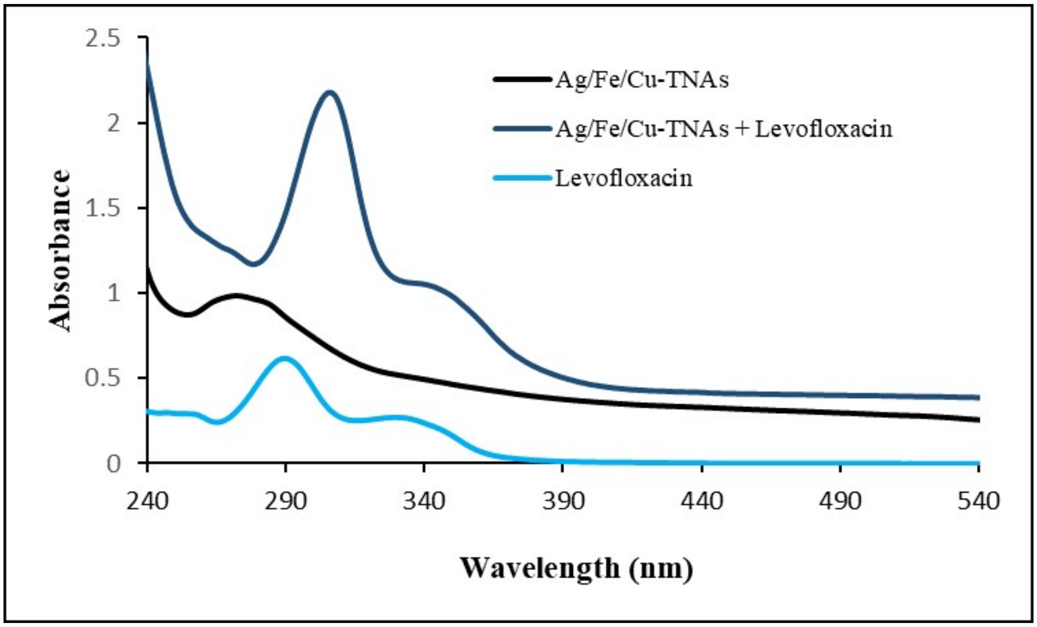

Fig. S2 Comparative UV-Visible spectra of Ag/Fe/Cu-TNAs, Levofloxacin and interaction between them

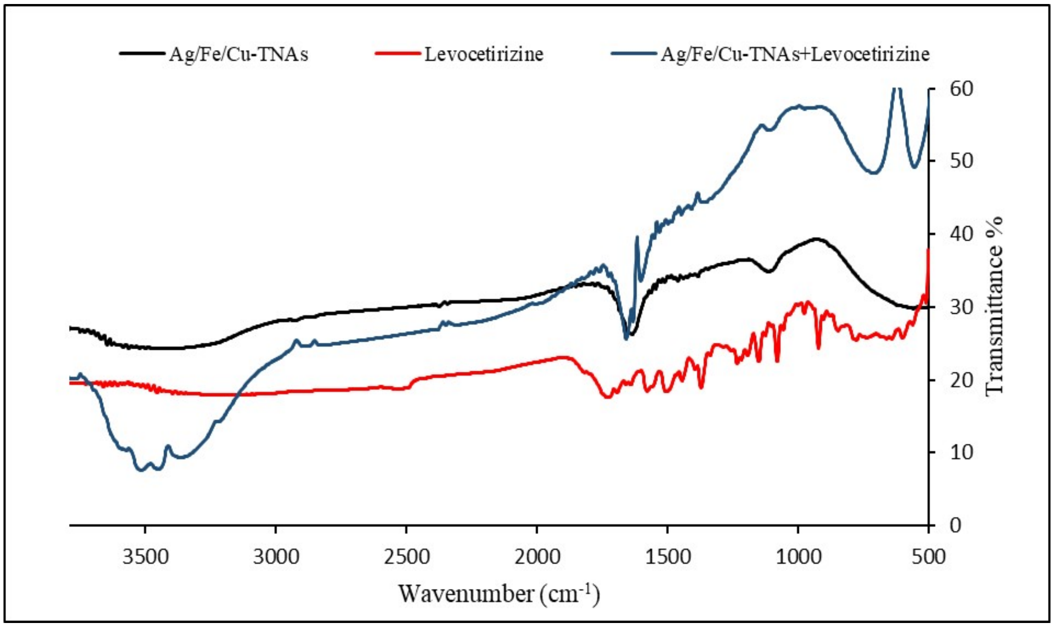

Fig. S3 Comparative FT-IR spectra of Ag/Fe/Cu-TNAs, Levocetirizine and interaction between them

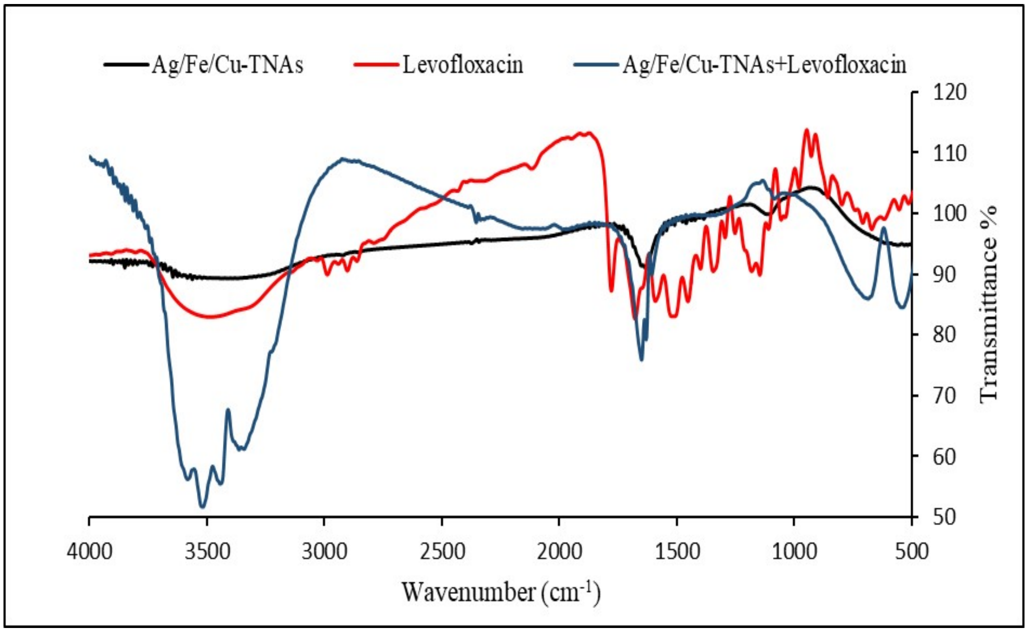

Fig. S4 Comparative FT-IR spectra of Ag/Fe/Cu-TNAs, Levocetirizine and interaction between them

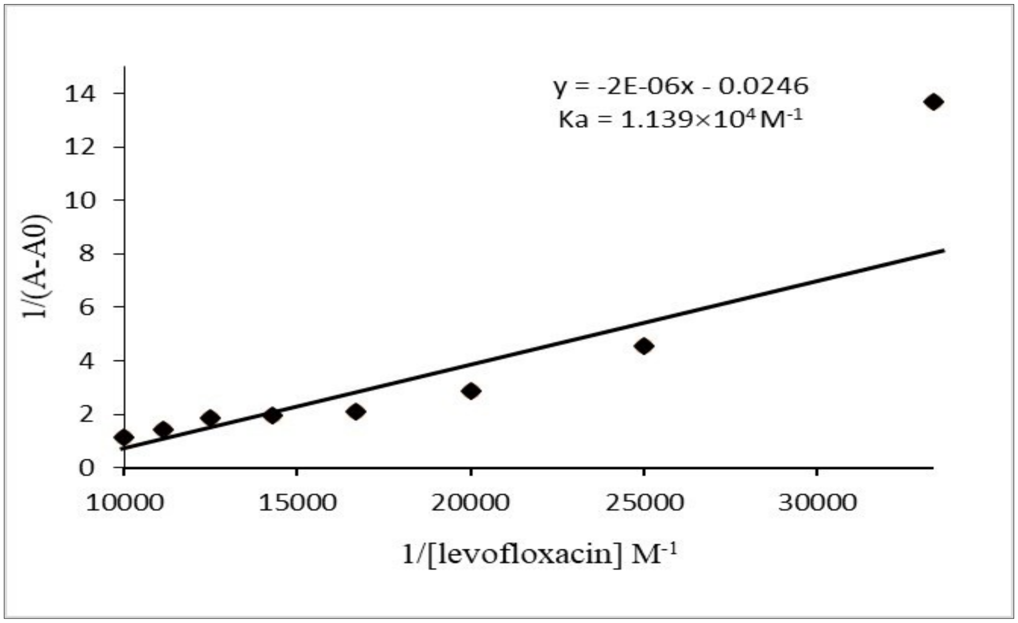

Fig. S5 Binding constant for the interaction between levofloxacin and Ag/Fe/Cu-TNAs measured by using Benesi Hildebrand equation

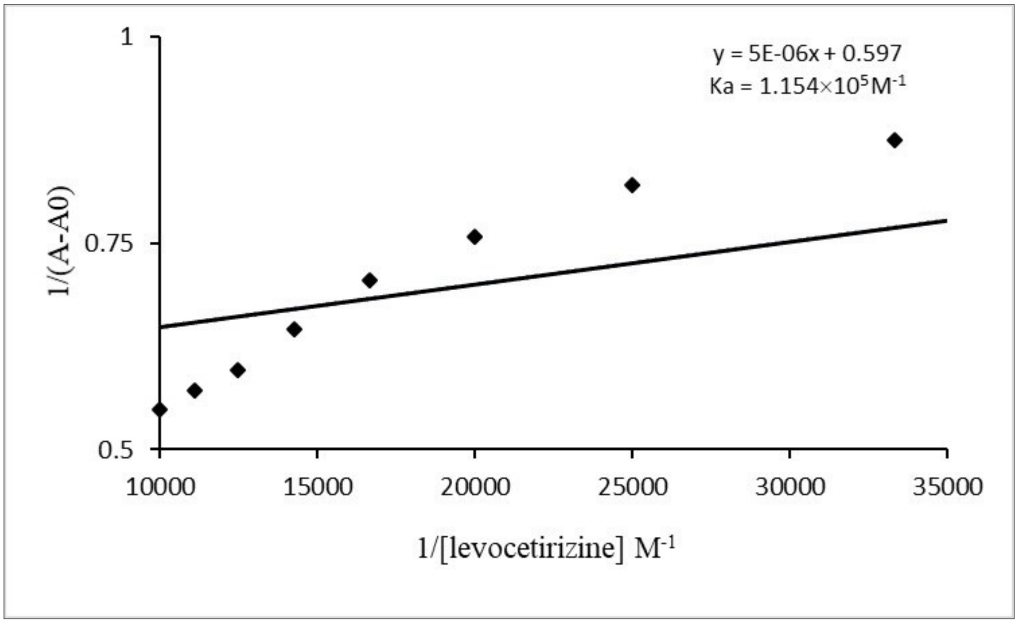

Fig. S6 Binding constant for the interaction between levocetirizine and Ag/Fe/Cu-TNAs measured by using Benesi Hildebrand equation

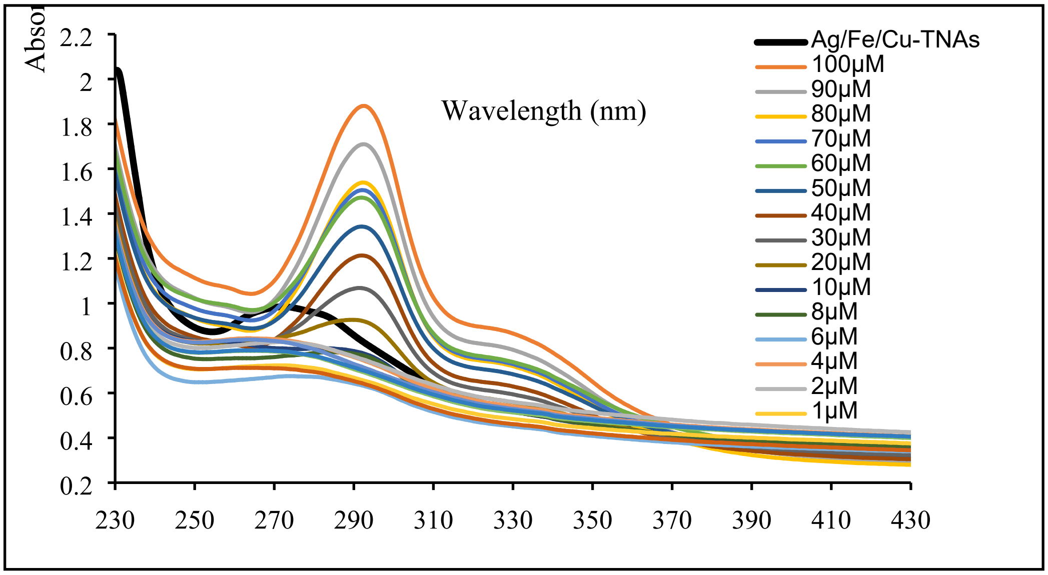

Fig. S7 Absorbance of levofloxacin-Ag/Fe/Cu-TNAs assembly at decreasing concentrations

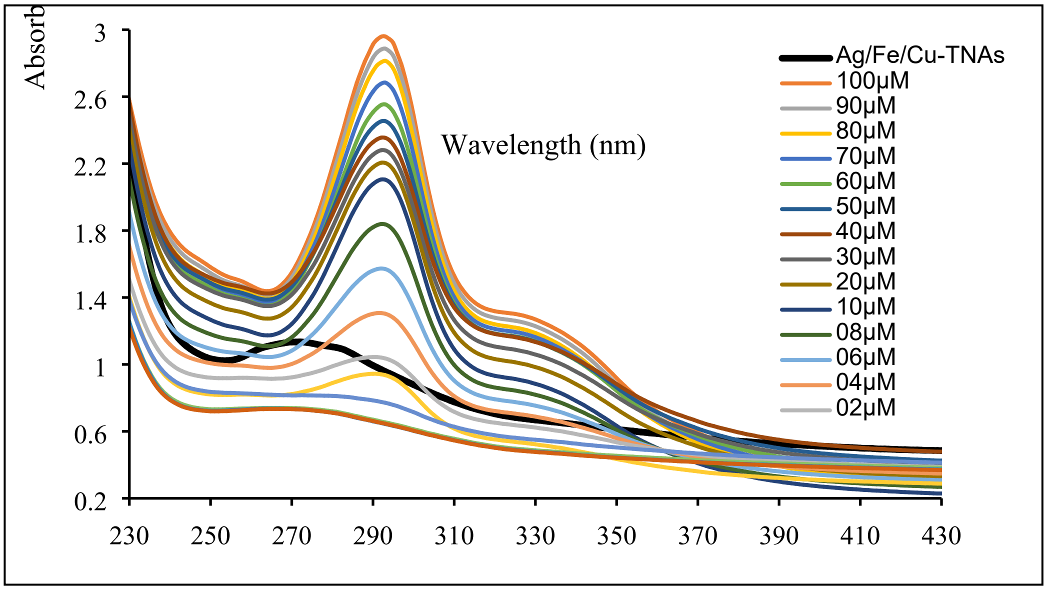

Fig. S8 Absorbance of levocetirizine-Ag/Fe/Cu-TNAs assembly at decreasing concentrations

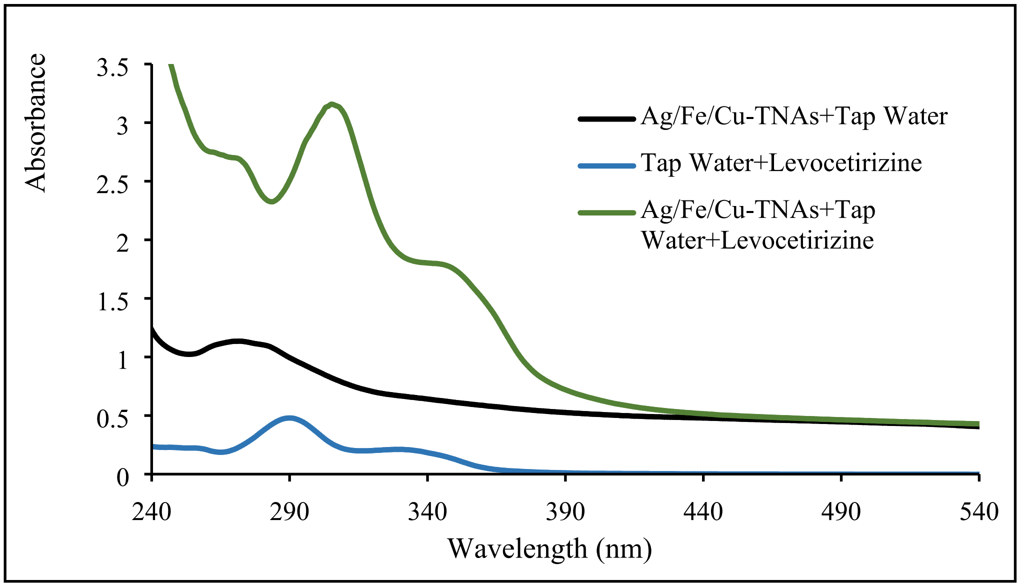

Fig. S9 Detection of levocetirizine in tap water

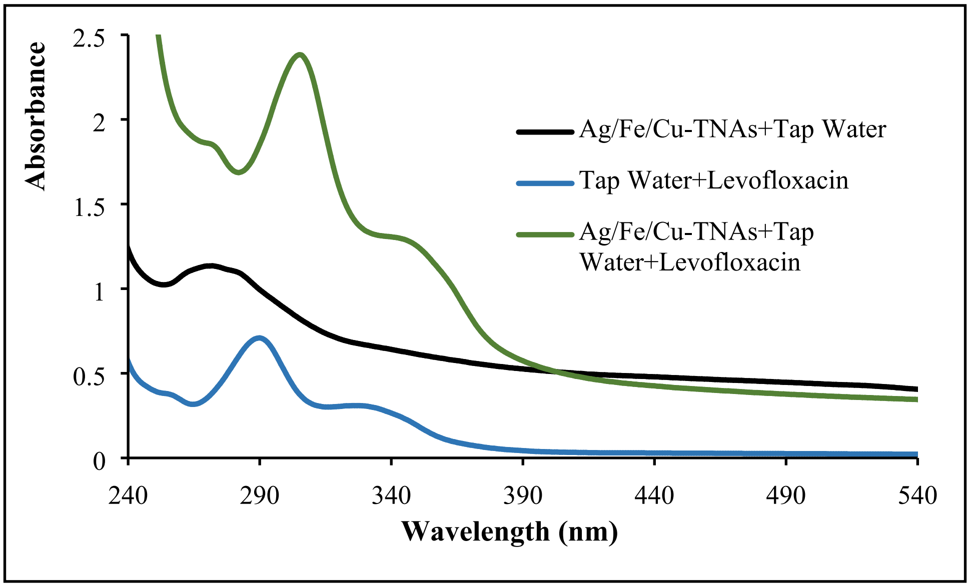

Fig. S10 Detection of levofloxacin in tap water

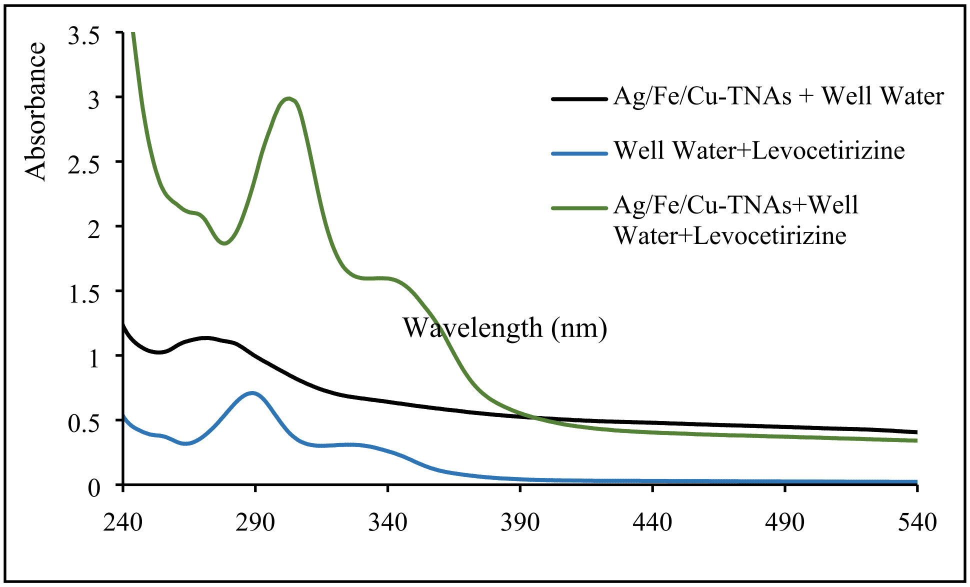

Fig. S11 Detection of levocetirizine in well water

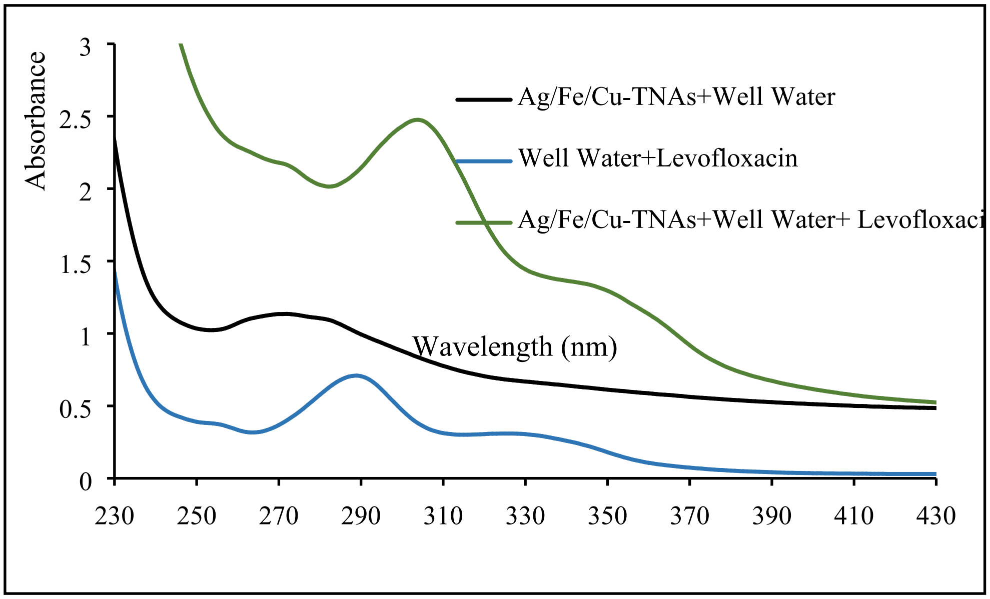

Fig. S12 Detection of levofloxacin in well water

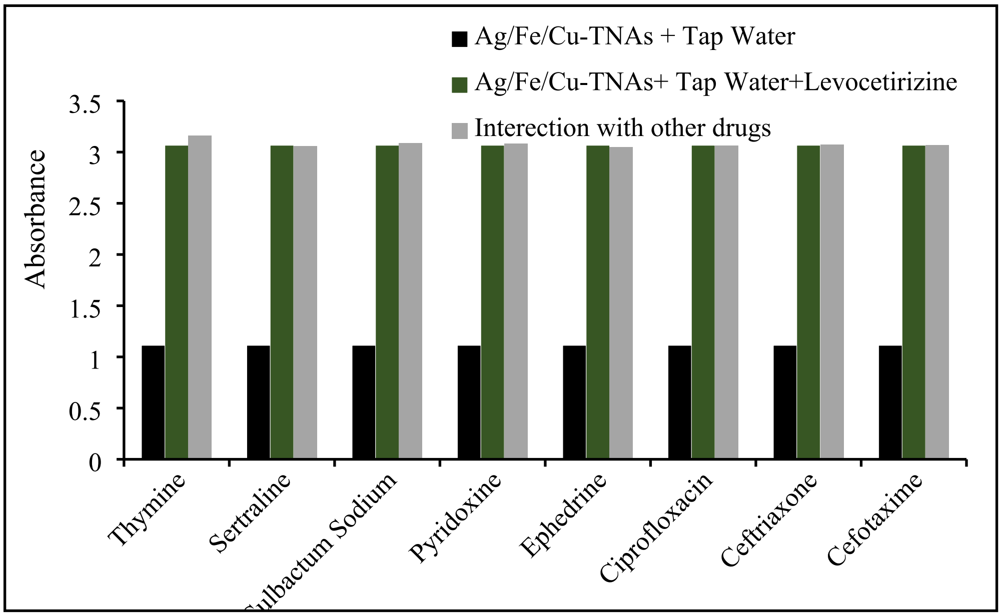

Fig. S13 Selectivity analysis for levocetirizine in tap water by Ag/Fe/Cu-TNAs

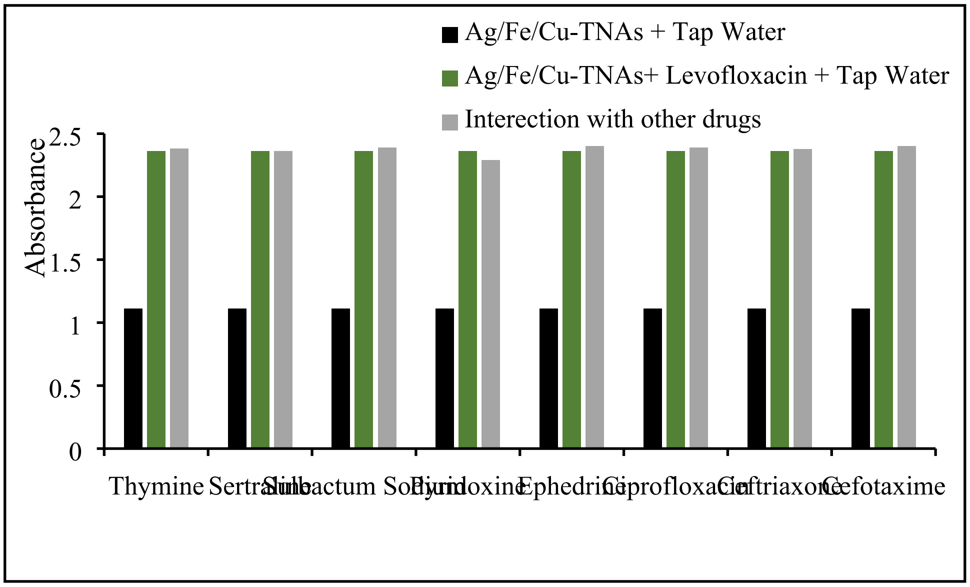

Fig. S14 Selectivity analysis for levofloxacin in tap water by Ag/Fe/Cu-TNAs

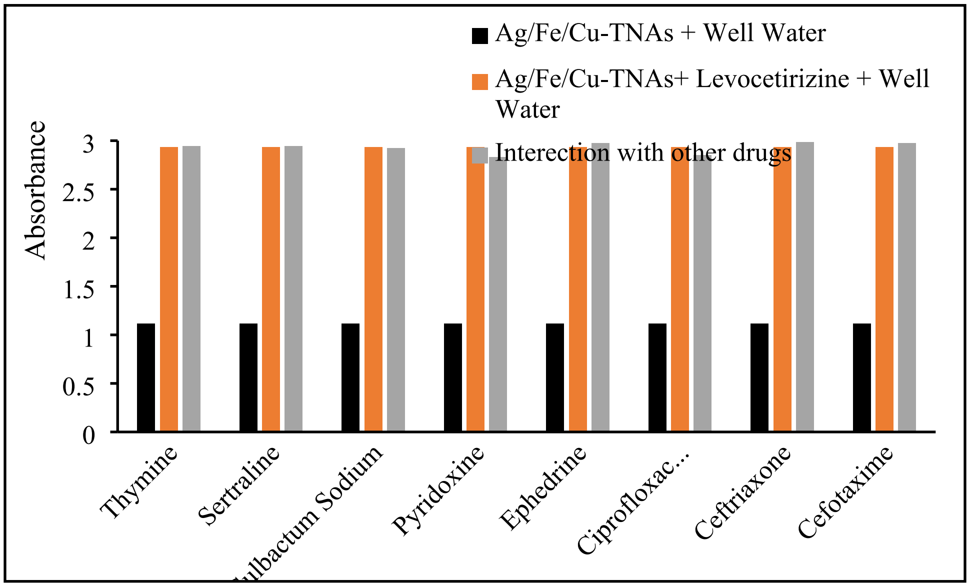

Fig. S15 Selectivity analysis for levocetirizine in well water by Ag/Fe/Cu-TNAs

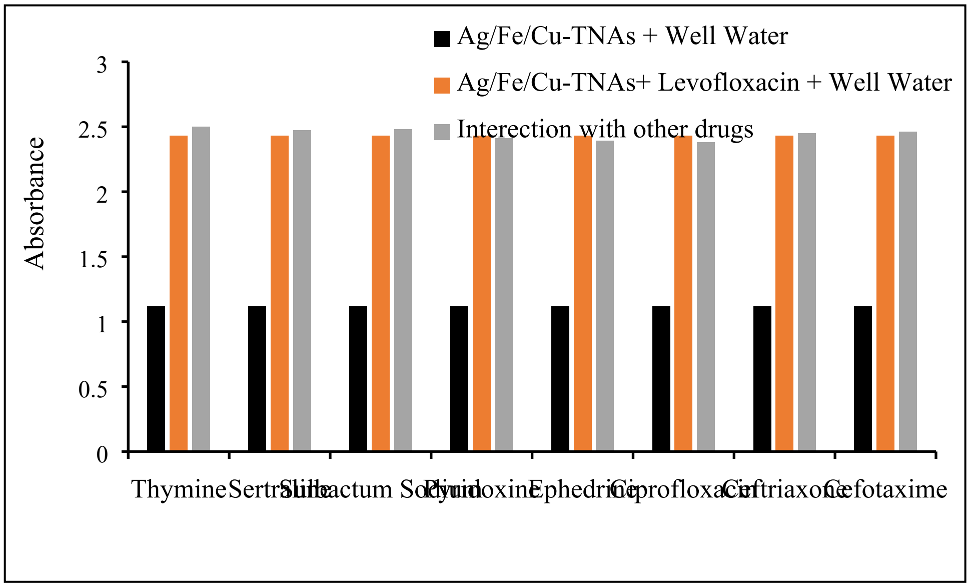

Fig. S16 Selectivity analysis for levofloxacin in well water by Ag/Fe/Cu-TNAs

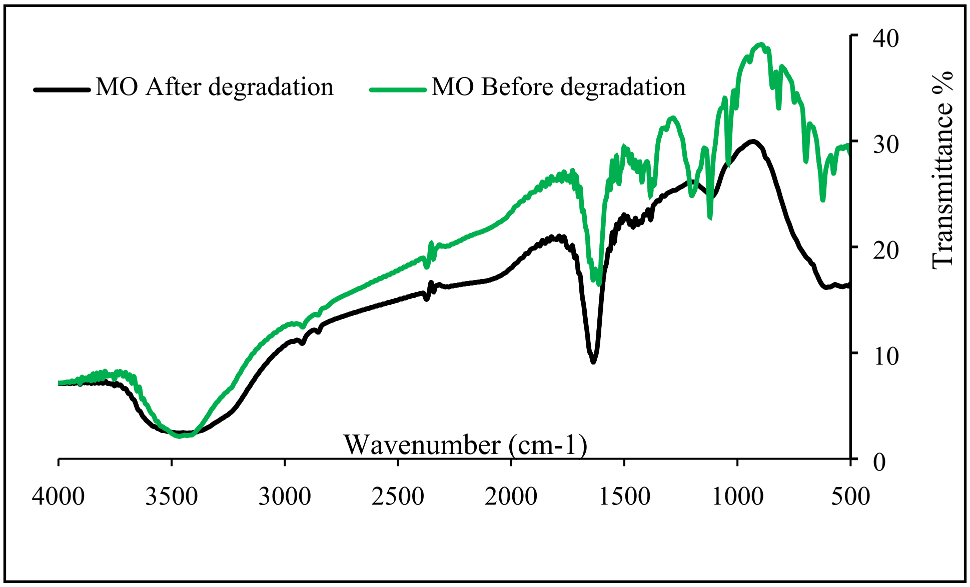

Fig. S17 Comparative FT-IR spectra of methyl orange before and after degradation in the presence of H_2O_2 and Ag/Fe/Cu-TNAs

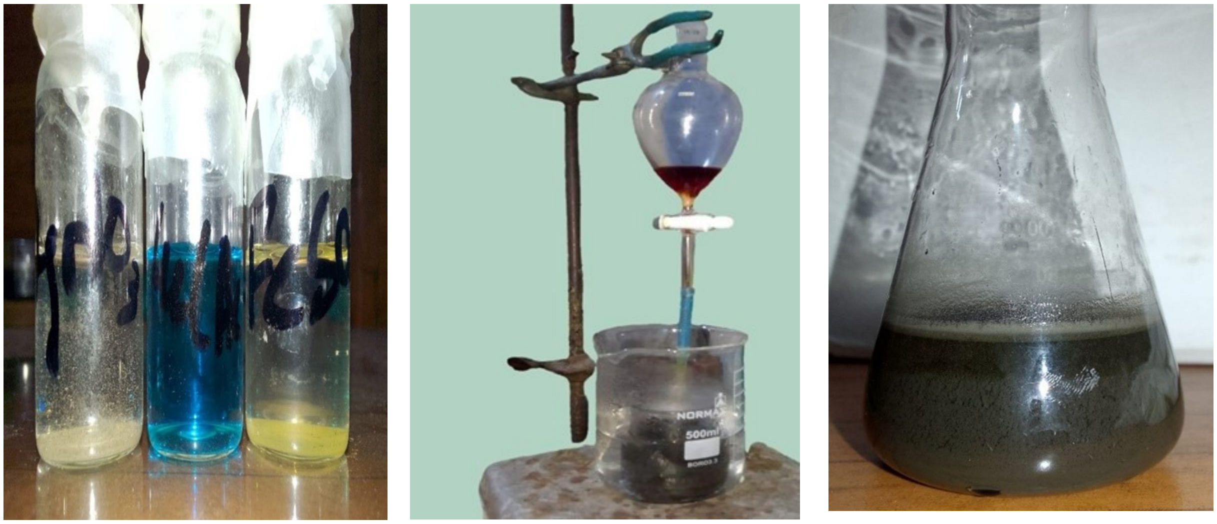

Fig. S18 a) Salts solutions **b)** addition of *Illicium verum* fruit extract and **c)** greyish green colored

Ag/Fe/Cu-TNAs
